# Biological and RNA regulatory function of MOV10 in mammalian germ cells

**DOI:** 10.1186/s12915-019-0659-z

**Published:** 2019-05-14

**Authors:** Kaiqiang Fu, Suwen Tian, Huanhuan Tan, Caifeng Wang, Hanben Wang, Min Wang, Yuanyuan Wang, Zhen Chen, Yanfeng Wang, Qiuling Yue, Qiushi Xu, Shuya Zhang, Haixin Li, Jie Xie, Mingyan Lin, Mengcheng Luo, Feng Chen, Lan Ye, Ke Zheng

**Affiliations:** 1 State Key Laboratory of Reproductive Medicine, Nanjing Medical University, Nanjing, 211166 China; 2 Department of Preventive Medicine, Heze Medical College, Heze, 274000 China; 3 School of Basic Medical Sciences, Nanjing Medical University, Nanjing, 211166 China; 4 School of Basic Medical Sciences, Wuhan University, Wuhan, 430072 China; 5 Department of Forensic Medicine, Nanjing Medical University, Nanjing, 211166 China

**Keywords:** MOV10, MOV10L1, RNA-binding protein, RNA helicase, miRNA, piRNA, Splicing, Testis, Male germ cells, Spermatogonia

## Abstract

**Background:**

RNA regulation by RNA-binding proteins (RBPs) involve extremely complicated mechanisms. MOV10 and MOV10L1 are two homologous RNA helicases implicated in distinct intracellular pathways. MOV10L1 participates specifically in Piwi-interacting RNA (piRNA) biogenesis and protects mouse male fertility. In contrast, the functional complexity of MOV10 remains incompletely understood, and its role in the mammalian germline is unknown. Here, we report a study of the biological and molecular functions of the RNA helicase MOV10 in mammalian male germ cells.

**Results:**

MOV10 is a nucleocytoplasmic protein mainly expressed in spermatogonia. Knockdown and transplantation experiments show that MOV10 deficiency has a negative effect on spermatogonial progenitor cells (SPCs), limiting proliferation and in vivo repopulation capacity. This effect is concurrent with a global disturbance of RNA homeostasis and downregulation of factors critical for SPC proliferation and/or self-renewal. Unexpectedly, microRNA (miRNA) biogenesis is impaired due partially to decrease of miRNA primary transcript levels and/or retention of miRNA via splicing control. Genome-wide analysis of RNA targetome reveals that MOV10 binds preferentially to mRNAs with long 3′-UTR and also interacts with various non-coding RNA species including those in the nucleus. Intriguingly, nuclear MOV10 associates with an array of splicing factors, particularly with SRSF1, and its intronic binding sites tend to reside in proximity to splice sites.

**Conclusions:**

These data expand the landscape of MOV10 function and highlight a previously unidentified role initiated from the nucleus, suggesting that MOV10 is a versatile RBP involved in a broader RNA regulatory network.

**Electronic supplementary material:**

The online version of this article (10.1186/s12915-019-0659-z) contains supplementary material, which is available to authorized users.

## Background

Germ cell differentiation in the testis involves a precisely orchestrated developmental program that originates from spermatogonial progenitor cells (SPCs) and proceeds through mitotic proliferation, meiotic division, and post-meiotic differentiation to produce haploid spermatozoa. Recent studies have shown that the transcriptome of the mammalian testis is exceptionally complex [1], due to the involvement of a vast variety of non-coding RNAs (ncRNAs) [2, 3]. Regulatory RNA species expressed in the mammalian testis encompass microRNAs (miRNAs), Piwi-interacting RNAs (piRNAs), and long non-coding RNAs (lncRNAs). MiRNAs (21–23 nt in length) are versatile regulators of gene expression. Similar to protein-coding mRNAs, testicular miRNAs are expressed in a cell- and developmental-specific manner and function post-transcriptionally to control the stability and/or translation of their target mRNAs [4, 5]. MiRNA biogenesis typically starts with transcription of long hairpin-embedded primary transcripts (pri-miRNAs) that are successively cleaved by two enzymes, Drosha in the nucleus and Dicer in the cytoplasm, to produce short stem-loop precursors (pre-miRNAs) and miRNA duplexes, respectively. Following unwinding of these duplexes, Argonaute proteins bind to single-stranded mature miRNAs and assemble into RNA-induced silencing complexes (RISC) to execute gene silencing [6, 7]. Noteworthy, the cataloging of the miRNA-like function of piRNAs (24–31 nt in length) is also emerging [8–10]. PiRNAs are a germline-specific class of small RNAs that are required for de novo DNA methylation and repression of retrotransponsons [11–13]. Long single-stranded piRNA precursors are transcribed from piRNA clusters, directed to the primary piRNA pathway, which may couple with a secondary amplification loop, and are processed into mature piRNAs through a series of cleavage, trimming, and modification steps. A third major class of regulatory RNA species, lncRNAs, are defined as non-protein coding transcripts (> 200 nt in length). LncRNAs fulfill a wide variety of regulatory roles dictated by their RNA structure and chemistry [14, 15]. Mammalian male germ cells express thousands of unique lncRNAs in developmental-specific patterns [3, 16], and the regulatory function of a subset has been characterized [17–20]. Primary precursor transcripts for small ncRNAs, such as miRNAs and piRNAs, are often considered lncRNAs that serve as substrates for enzymes [21]. LncRNAs are expressed at much lower levels than transcripts of protein-coding genes, limiting comprehensive detection by standard approaches [15, 22].

RNA species in vivo are usually bound by RNA-binding proteins (RBPs), forming ribonucleoprotein particles (RNPs). Germ cells express high levels of RBPs to enable dynamic control of both mRNAs and ncRNAs. Loss-of-function studies have shown that many different RBPs are involved in multiple aspects of RNA metabolism, including RNA processing and splicing, and are essential for the completion of spermatogenesis. Splicing of protein-coding genes is a dynamically RBP-regulated RNA processing process [23] and widely integrated with other layers of gene expression control in the nucleus [24]. Emerging evidence shows that splicing may crosstalk with the biogenesis or metabolism of various non-coding RNA species [25–30]. Our understanding of miRNA biogenesis is expanding because non-canonical pathways including those dependent on splicing but independent of Drosha or Dicer, such as mirtron (short hairpin pre-mRNA), are also emerging [31–33].

The RNA helicase MOV10L1 (Moloney leukemia virus 10 like-1) was initially identified as a testis-specific gene [34]. MOV10L1 associates with Piwi proteins and is a master regulator in piRNA biogenesis [35–38]. Disruption of MOV10L1 helicase activity leads to the loss of pre-pachytene piRNAs, resulting in activation of retrotransposons, early meiotic arrest, and male infertility [35, 37, 39]. MOV10L1 is also required for the biogenesis of pachytene piRNAs, a yet poorly understood piRNA class that is essential for the maintenance of genomic integrity in post-meiotic male germ cells [36]. MOV10L1 harbors bona-fide RNA helicase activity and is proposed to unwind RNA secondary structures to facilitate the endonucleolytic cleavage of primary piRNA precursors [38]. Of note, MOV10L1 has a paralogue MOV10. These two helicases likely reflect vertebrate counterparts that evolved from a common ancestor, the DExD-box protein *Drosophila* Armitage [40]. MOV10 inhibits retroviral replication and was initially discovered in a mouse strain in which a single proviral copy of Moloney leukemia virus (MLV) was integrated into the *Mov10* locus [41–43]. Similar to MOV10L1, MOV10 belongs to the UPF1-like helicase superfamily 1 (SF1) and harbors 5′ to 3′ RNA helicase activity [38, 44]. MOV10 interacts with LINE1 RNPs and inhibits retroelements [45, 46] and also regulates mRNA stabilization and/or translation by targeting 3′-UTRs [44, 47, 48]. In contrast to MOV10L1, MOV10 interacts with the miRNA machinery and is involved in miRNA-mediated post-transcriptional regulation [47–50]. Most studies in non-germ cells have shown that MOV10 localizes to the RNA processing body (P body) in the cytoplasm. As a few exceptions, nuclear localization of endogenous MOV10 has also been observed in human cell lines and postnatal mouse brain [51–53]. Currently, the role of MOV10 in the mammalian testis is unknown.

Here, we have characterized the MOV10 RNA targetome in postnatal mouse testis using high-throughput sequencing of RNA isolated by crosslinking immunoprecipitation (HITS-CLIP) and its protein interactome using immunoprecipitation followed by mass spectrometry (IP-MS). Our results reveal a previously uncharacterized nuclear role of MOV10 in miRNA processing and splicing. These profiling and analytical data, together with functional studies, demonstrate that MOV10 regulates fate decisions and shapes transcriptome in germ cell progenitors.

## Results

### MOV10 is a nucleocytoplasmic protein in the testis and mainly expressed in spermatogonia

**Fig. 1:**
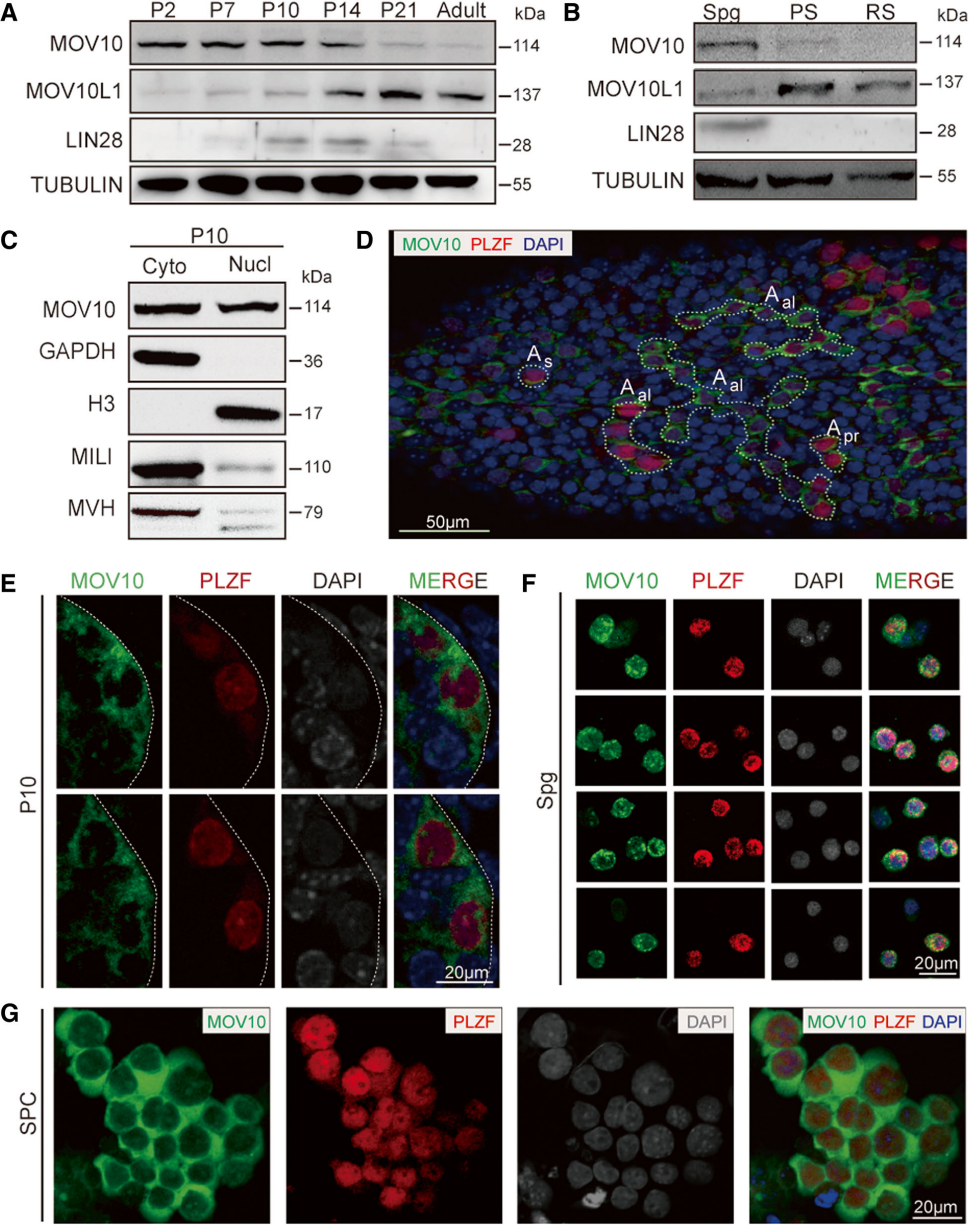
Expression and localization patterns of MOV10 in male germ cells. **a** Time-course western blot analysis of MOV10 in postnatal mouse testes, compared with MOV10L1 and LIN28. TUBULIN was used as a loading control. Mouse testes were collected from P2 through adulthood. **b** Expression patterns of MOV10, MOV10L1, and LIN28 in different populations of male germ cells. Spg, spermatogonia; PS, pachytene spermatocytes; RS, round spermatids. **c** Western blot analysis of MOV10 in cytoplasmic and nuclear fractions of P10 testis. GAPDH and H3 were used as cytoplasmic and nuclear markers, respectively. MILI and MVH are known to be present in perinuclear granules in the cytoplasm. **d** Whole-mount immunostaining for MOV10 and PLZF in seminiferous tubules. Image shows A_s_, A_pr_, and A_al_ (A4, A8, A16) clusters of spermatogonia that are interconnected by intercellular cytoplasmic bridges due to incomplete cytokinesis. Dotted lines mark clusters of PLZF (shown in red) and MOV10 (shown in green) double-positive cells, counterstained with DAPI (shown in blue). Scale bars, 50 μm. **e**–**g** Frozen sections of testes from P10 (**e**), Spg isolated from P6–8 testes (**f**), and in vitro cultured SPCs (**g**) were co-immunostained for MOV10 and PLZF. Panel **e** shows high-magnification images of regions marked by dashed lines. Scale bar, 20 μm

We first characterized MOV10 expression in postnatal testis using anti-MOV10 antibody (Additional file 1: Figure S1 and Methods: MOV10 Antibody Validation). MOV10 protein was present in postnatal day 2 (P2) to P14 testis, but weakly expressed in P21 and adult testis (Fig. 1a). P10 testis, with abundance of the spermatogonia marker LIN28 [54], contained relatively high levels of MOV10 protein. This contrasts with the known expression pattern of MOV10L1, which increases significantly at P14 and peaks at P21, concomitant with the appearance of pachytene spermatocytes [35, 37]. We next characterized MOV10 protein levels in isolated spermatogenic populations, including purified spermatogonia (80%; Additional file 1: Figure S2), pachytene spermatocytes (80%; Additional file 1: Figure S2), and round spermatids (90%; Additional file 1: Figure S2), and found that MOV10 was most abundant in spermatogonia (Fig. 1b). Western blot analysis of MOV10 protein in subcellular fractions prepared from P10 testes confirmed the presence of considerable amounts of MOV10 protein in the nuclear fraction (Fig. 1c), which contained only minimal levels of MILI and MVH, well-defined piRNA factors that mainly associate with cytoplasmic perinuclear granules called nuage [55]. The nuclear fraction therefore contained only minimal contamination with components associated with the nuclear membrane. Co-immunostaining for MOV10 and PLZF, a well-established marker for spermatogonia, showed that MOV10 was predominantly expressed in PLZF-positive spermatogonial clusters in whole-mount adult testis tissue (Fig. 1d) and in DAPI-faint, PLZF-positive spermatogonia in P10 testis sections (Fig. 1e). Consistent with our western blot analyses, MOV10 was primarily cytoplasmic but with punctate staining in the nucleus in P10 testis (Fig. 1e), in spermatogonia isolated from P6–8 testes (Fig. 1f) and in in vitro cultured spermatogonial progenitor cells (SPCs) (Fig. 1g). We also detected a strong dot-like nuclear signal of MOV10 in embryonic day 16.5 (E16.5) gonocytes and in P1 testis (Additional file 1: Figure S1). These findings establish MOV10 as a nucleocytoplasmic protein in early male germ cells.

### MOV10 enhances in vitro proliferation and in vivo repopulation capacity of spermatogonial progenitor cells

**Fig. 2:**
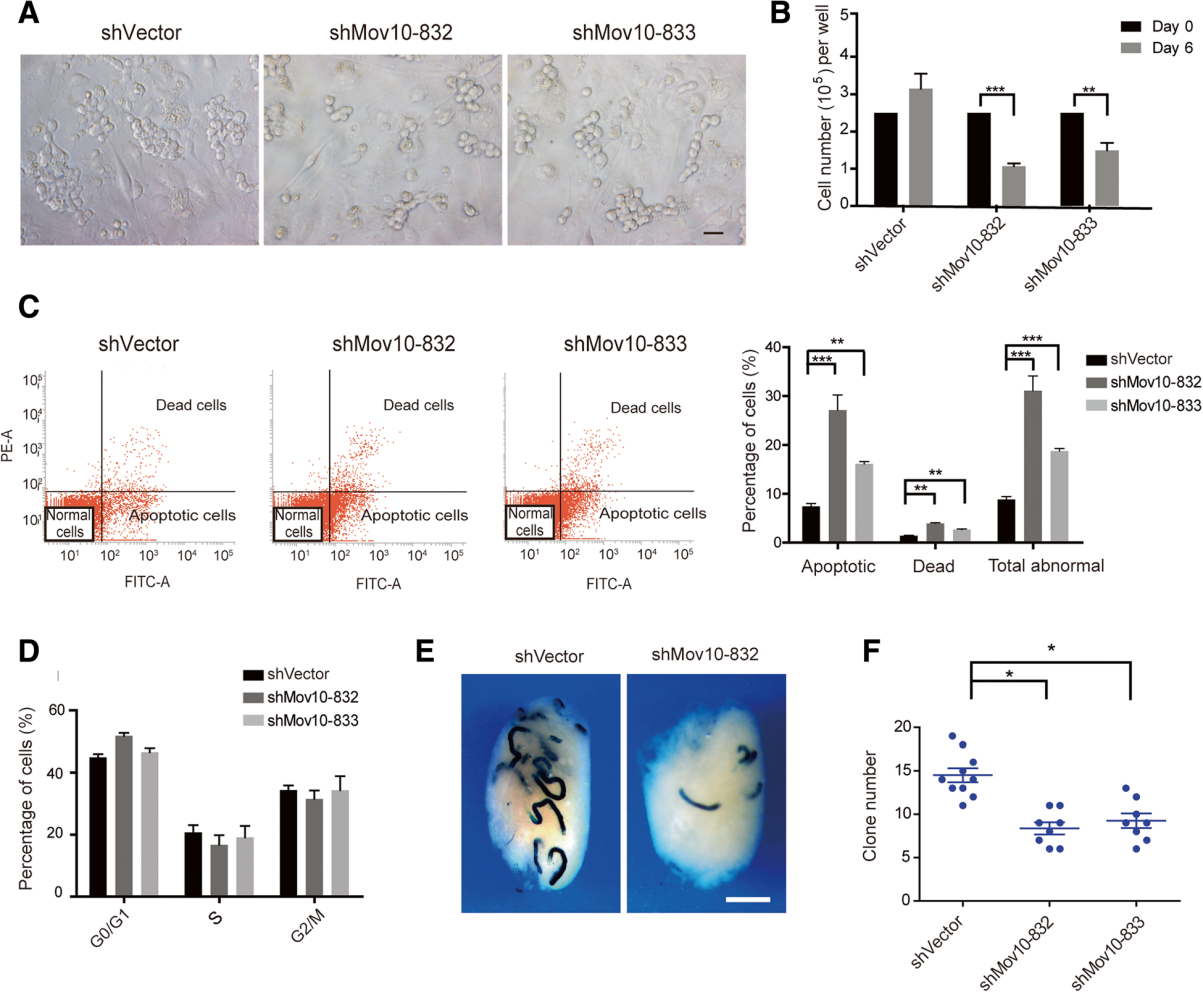
*Mov10* knockdown affects cell fate decisions in SPC. **a** Morphological differences between *Mov10* shRNA (shMov10-832 or shMov10-833) and control (shVector) virus transduced SPC cultures at 6 days following lentiviral transduction. *Mov10* shRNA cultures exhibit dissociation of clump-forming cells and contain only few and small grape-shaped colonies. Scale bar, 20 μm. **b** Significantly lower cell numbers in SPC cultures transduced with *Mov10* shRNA vs cells transduced with control virus. **c**, **d** Flow cytometric analysis of apoptosis (**c**) and cell cycle (**d**). The error bars in **b**, **c**, and **d** represent variation (SEM) among biological triplicates. **e**, **f** Reduced repopulation capacity of SPCs after *Mov10* knockdown. *Mov10* shRNA or control vector transduced cells were transplanted into total 13 recipient testes. Donor-derived spermatogenic colonies (beta-glucuronidase transgene positive) were visualized by X-gal staining and counted. Scale bar, 2 mm. *p < 0.05; **p < 0.01; ***p < 0.001

To investigate the physiological function of MOV10 in male germ cells, we knocked down *Mov10* in in vitro cultured SPCs using small hairpin RNAs (shMov10-832 and shMov10-833) designed against *Mov10* sequences with low homology to *Mov10l1* (Additional file 1: Figure S3). The off-target analysis of both shRNA sets using the Sylamer program revealed the potential trend to influence other genes was very low (Additional file 1: Figure S4) [56]. Transduction of SPCs with a lentivirus encoding shMov10-832 or shMov10-833 resulted in a significant reduction of *Mov10* mRNA levels, whereas *Mov10l1* transcript levels were not affected (Additional file 1: Figure S3). Western blot analysis confirmed reduced MOV10 protein levels in transduced cells (Additional file 1: Figure S3). SPCs with shMov10-mediated knockdown of *Mov10* formed smaller cell clusters than control cultures, and clusters lacked the typical grape-shaped appearance (Fig. 2a). Furthermore, SPC cultures subject to *Mov10* knockdown contained significantly lower cell numbers than control cultures after 6 days of culture (Fig. 2b). Flow cytometric studies showed that SPC cultures with shMov10-mediated knockdown of *Mov10* contained significantly more apoptotic and dead cells than control cultures (Fig. 2c) but did not significantly differ in respect to cell cycle progression (Fig. 2d).

To examine if *Mov10* knockdown affected the capacity of SPCs to restore spermatogenesis, we transplanted cell suspensions of control cultures and *Mov10* knockdown SPC cultures from Rosa transgenic mice into germ cell-depleted recipient testes in which endogenous spermatogenesis had been abolished. Identification of donor-derived spermatogenic colonies by X-gal staining revealed a diminished ability of *Mov10* knockdown cells to colonize recipient testes (Fig. 2e), with an approximately 50% decrease in colony numbers compared with control SPCs (Fig. 2f). We next assessed the effect of *Mov10* knockdown on the capacity of spermatogonial cells in vivo, lentivirus expressing shMov10 or shVector was prepared and subsequently transduced into adult mouse testes [57, 58]. The signal of EGFP was observed across spermatogenic cells at different developmental stages (Additional file 1: Figure S5), which indicates the successful infection of lentivirus. Numbers of spermatogonial cells positive for TUNEL were increased in the seminiferous tubules of mouse testes transduced with shMov10 in comparison to those with shVector (Additional file 1: Figure S5). Taken together, the outcomes of both in vitro and in vivo studies demonstrate that MOV10 plays a pivotal role in determining the SPC fate.

### MOV10 maintains gene expression and the miRNA pool

Transcript profiling by RNA deep sequencing (RNA-seq) showed that shMov10-mediated knockdown of *Mov10* in SPC was associated with up- and downregulation of 407 and 282 protein-coding genes, respectively (Additional file 1: Figure S6). Genes subject to downregulation in the absence of MOV10 included *Etv5*, *Bcl6b*, and *Zbtb16*, all of which encode factors necessary for SPC proliferation and/or self-renewal [59, 60]. In contrast, the significantly enriched terms among upregulated genes were related with ion transport and membrane potential (Additional file 1: Figure S7). Reverse transcription-quantitative real-time polymerase chain reaction (RT-qPCR) confirmed significantly lower *Etv5* and *Bcl6b* transcript levels in *Mov10* knockdown versus control SPC cultures, whereas *Zbtb16* mRNA levels were similar (Additional file 1: Figure S3). However, similar to ETV5 and BCL6B, ZBTB16 protein levels were substantially reduced in *Mov10* knockdown SPC vs controls (Additional file 1: Figure S3), suggesting that MOV10 regulates mRNA stability and/or translation efficiency, the latter of which is supported by observations that MOV10 associates with polysomes (Additional file 1: Figure S3). In contrast, MOV10L1 associated with RNPs but not with ribosomes or polysomes, implicating a functional non-redundancy of MOV10 and MOV10L1. Knockdown of *Mov10* in SPCs also produced changes in transcript levels of lncRNAs. Similar to mRNAs, affected lncRNA transcripts were predominantly upregulated (Additional file 1: Figure S6). Following overexpression of MOV10 in SPCs, we observed increased colony size and cell number vs controls, and upregulation of *Etv5*, *Bcl6b*, and *Zbtb16* mRNA or protein levels (Additional file 1: Figure S8). The opposite phenotypes associated with knockdown and overexpression of *Mov10* in SPC further validate its regulatory role on SPC proliferation and/or self-renewal.

**Fig. 3:**
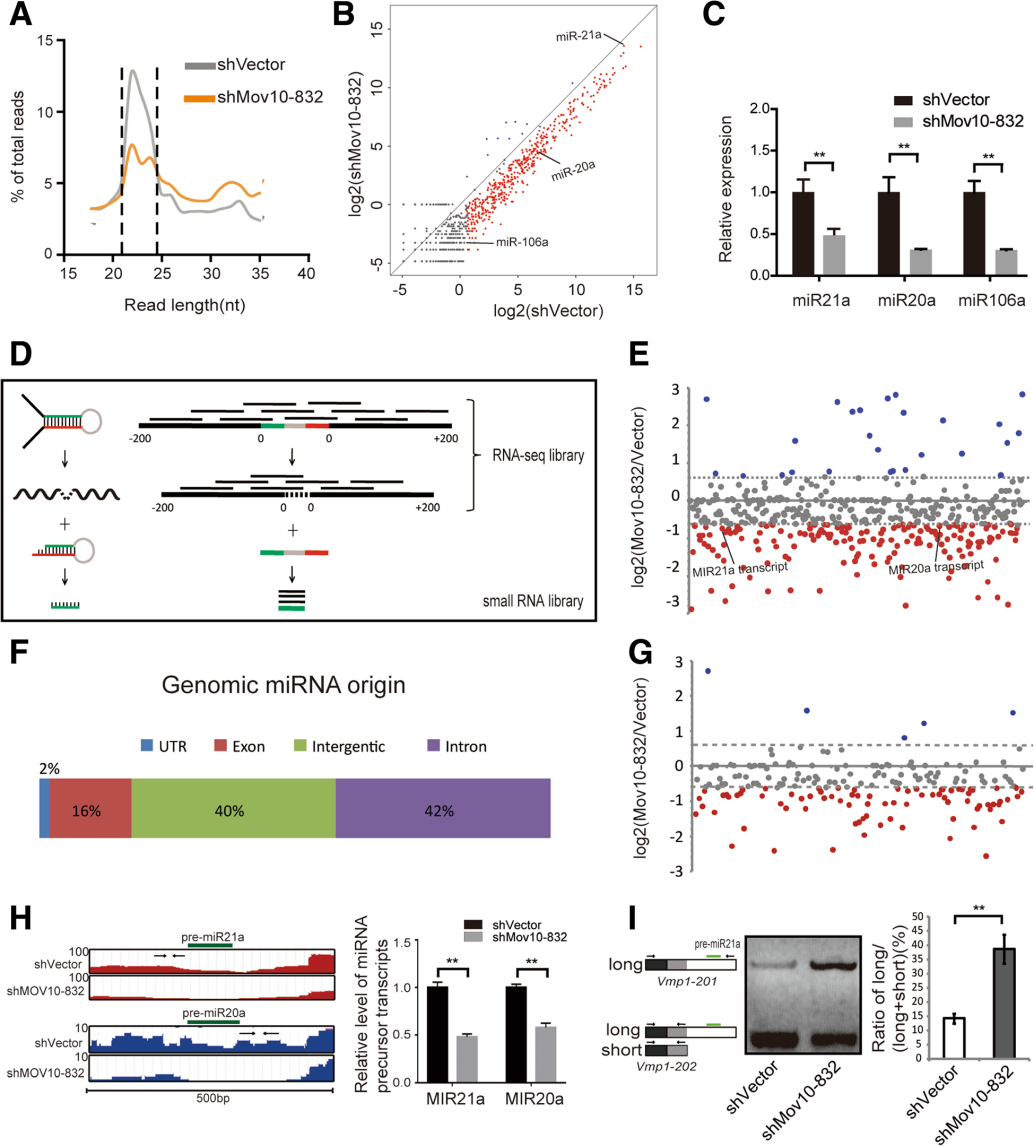
*Mov10* knockdown affects the production of miRNA. **a** Read length profile for small RNAs showing that the percentage of small RNA reads were downregulated after *Mov10* shRNA treatment. **b** Scatter plot of miRNA expression data from small RNA-seq represented as log2 values of *Mov10* knockdown versus control samples. **c** qPCR validation of selected miRNAs with well-known function in SPCs. **d** Schematic representation of a general step-wise miRNA processing from pri- to mature forms (left) and the corresponding read pick-up from RNA-seq library and small RNA library (right). See the rationale in the Methods. **e** Scatter plot showing change of the transcript of each downregulated miRNA represented as *Mov10* knockdown versus control samples. Results are mean values from three biological replicate RNA-seq libraries. **f** Distribution of downregulated miRNAs according to genomic origin of their mature form. **g** Analysis of intron-derived miRNAs using the same method as in panel E. **h** UCSC visualization of the genomic window of ± 200 nt flanking the hairpin of miR21a and miR20a shows lower RNA-seq read density in *Mov10* knockdown SPC versus vector control (left) and qPCR confirmed significant reduction of miR21a and miR20a primary transcripts (right). PCR primer sets are marked by arrows relative to the position to the pre-miRNA shown in green. **i** The long isoform *Vmp1-201* (ENSMUST00000018315.9) bearing the long 3′-UTR contains pre-miR21a (green line) and can be processed to produce the short isoform *Vmp1-202* (ENSMUST00000123590.7) and miR21a precursor for downstream miRNA processing (left). Exons (black box), the alternative part of 3′-UTR (white box) as well as the common part (gray box) of 3′-UTR are shown. Two different primer sets (arrows) were designed. The relative ratio of the long isoform over both (long+short) was quantified by Image J software (right)

Next, we evaluated the consequences of *Mov10* knockdown on small RNA levels. Read length profiling after small RNA sequencing revealed that 21–25 nt-long RNAs, which largely represent mature miRNAs, were particularly underrepresented in *Mov10* knockdown vs control SPCs (Fig. 3a). We found that inhibition of *Mov10* expression affected miRNA biogenesis globally, causing reduced levels of 446 miRNAs and upregulation of 10 miRNAs compared to control SPCs (> 1.5-fold and TPM > 1.5; Fig. 3b). RT-qPCR for selected miRNAs, including miR21a, miR20a, and miR106a, which play a critical role in determining SPC fate [61, 62], confirmed significant reduction of mature miRNA levels in *Mov10* knockdown vs control SPCs (Fig. 3c). Nevertheless, mRNA transcripts of *Drosha*, *Dgcr8*, and *Dicer* expressed similar levels in SPCs with *Mov10* knockdown compared with control cells (Additional file 1: Figure S6). Moreover, the protein levels of the above classical factors for miRNA biogenesis remained unchanged (Additional file 1: Figure S6). We then examined the transcript levels of the 446 downregulated miRNAs by designing a RNA-seq pipeline based on the principles of miRNA processing and RNA-seq (Fig. 3d). RNA-seq read density within an adjacent genomic window (± 200 nt) flanking the miRNA hairpin represents the whole level of miRNA transcript (i.e., combined levels of pri-miRNA and its miRNA-truncated fragments after initial processing) and the maximal potential of miRNA generation. The result showed an overall trend towards downregulation of miRNA transcripts. Specifically, the transcripts of 198 miRNAs were downregulated and transcripts of 34 miRNAs were upregulated (> 1.5-fold, Fig. 3e). Of 446 downregulated miRNAs, 189 were of intronic origin (Fig. 3f), and of these, 95 and 5 were downregulated and upregulated, respectively (> 1.5-fold, Fig. 3g). These bioinformatics analyses matched the read density distribution in genome browser visualization such as for miR20a and miR21a (Fig. 3h, left) and were also validated by RT-qPCR (Fig. 3h, right). These results indicate that downregulation of miRNAs may be attributed partly to attenuation of the transcription and/or stability of their transcripts.

### MOV10 regulates miRNA precursors via 3′-UTR processing and splicing

**Fig. 4:**
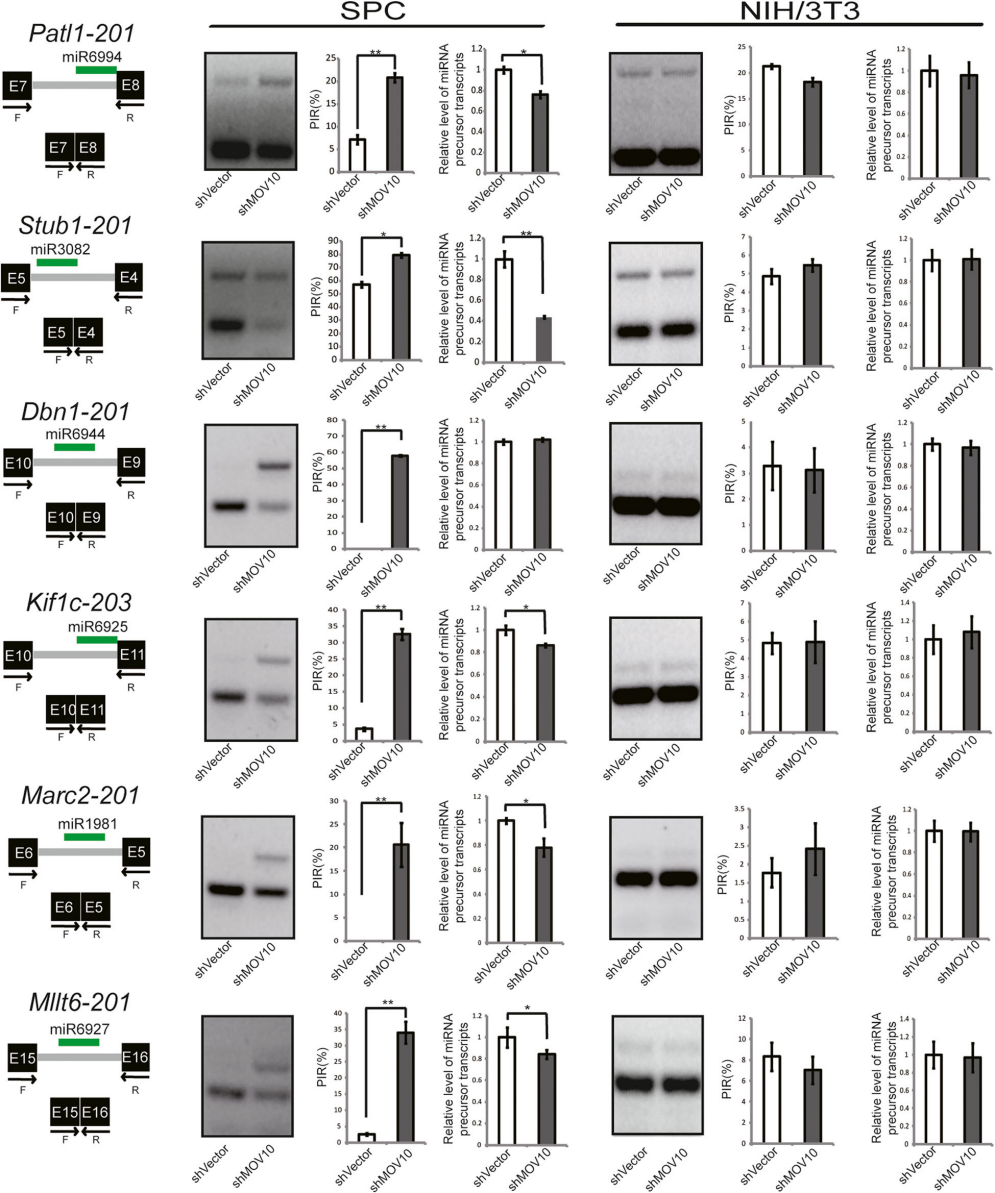
MOV10 regulates mirtron splicing in SPCs. PCR test of mirtron splicing events in SPCs and NIH/3 T3 cells. Total RNA samples from *Mov10* knockdown versus control SPCs were examined by semi-quantitative RT-PCR analyses. The diagrams depict two splicing isoforms, black boxes represent exons, gray lines represent introns, green lines represent miRNAs, and arrows mark the positions of forward (F) and reverse (R) primers. E, exon number. The percent of intron retention (PIR) value, used as an indication for the extent of alteration of a splicing event, was determined as the proportion of the intron retention isoform versus the total level of intron retention and intron exclusion isoforms. The change of miRNA transcripts was calculated as the relative whole level of intron retention and intron exclusion isoforms in *Mov10* knockdown over control. PCR products were quantified by Image J software. Results represent data from biological triplicates

The level of mature miRNAs depends on both the transcription rate and by posttranscriptional regulation which may bias splicing or processing of miRNA transcripts towards excision or retention of the miRNA, producing different levels of mature miRNAs from the same amount of precursor. Specifically, miR21a is located within 3′-UTR of the vacuole membrane protein-1 (*Vmp1*) gene [63]. Knockdown of *Mov10* in SPCs significantly altered the ratio of *Vmp1* transcripts with a long pri-miR21a containing 3′-UTR to all variants (Fig. 3i, middle and right). It suggests that this long 3′-UTR processing is regulated by MOV10, which is required for excision of miR21a precursor from the long isoform. More examples were provided for the role of MOV10 in 3′-UTR processing of miRNAs, including miR331, miR1191, and miR7673 (Additional file 1: Figure S9). We next analyzed splicing events in the primary transcripts of intronic miRNAs that were downregulated in SPCs after *Mov10* knockdown. We filtered at a lower stringency (> 1.5-fold and TPM > 1) to identify more intronic miRNAs potentially regulated by MOV10 and obtained 235 downregulated intronic miRNAs, of which 18 belonged to known mirtrons from a mouse mirtron database [64]. This database contains confirmed and a large proportion (ca. 50%) of predicted mirtrons. Of 18 tested known mirtron splicing events, 14 events were significantly changed upon *Mov10* knockdown (Fig. 4 and Additional file 1: Figure S10). In all cases, mirtrons were retained in the pre-mRNA holders, associated with lower mirtron transcript levels in a subset. NIH3T3, distinct from SPC, is a commonly used mouse cell line suitable for *Mov10* knockdown experiments and evaluation of gene expression using the same set of mouse-specific primers. In parallel, we examined 6 mirtron splicing events in NIH3T3 cell lines and found no significant change in the *Mov10* knockdown versus control samples (Fig. 4), suggesting a cell-type-dependent role of MOV10 for splicing regulation. In addition, of 14 tested splicing events of intronic miRNAs other than mirtrons, 4 events were significantly biased towards miRNA retention after *Mov10* knockdown in SPCs (Additional file 1: Figure S11). The crosstalk between pre-mRNA splicing and mirtron precursor processing prompted us to further examine the splicing regulatory role of MOV10 at a transcriptome-wide level. From RNA-seq analyses, many alternative splicing events were altered in *Mov10* knockdown cells (Additional file 1: Figure S12). Although *Mov10* knockdown was not associated with changes in the levels of mRNAs of various known splicing regulatory genes (Additional file 1: Figure S6), the involvement of MOV10 in splicing regulatory network has been implicated in a high-profile resource article [23], suggesting that MOV10 may play a direct nuclear role in splicing regulation.

### In vivo capture of RNA targets of MOV10 from juvenile testes

**Fig. 5:**
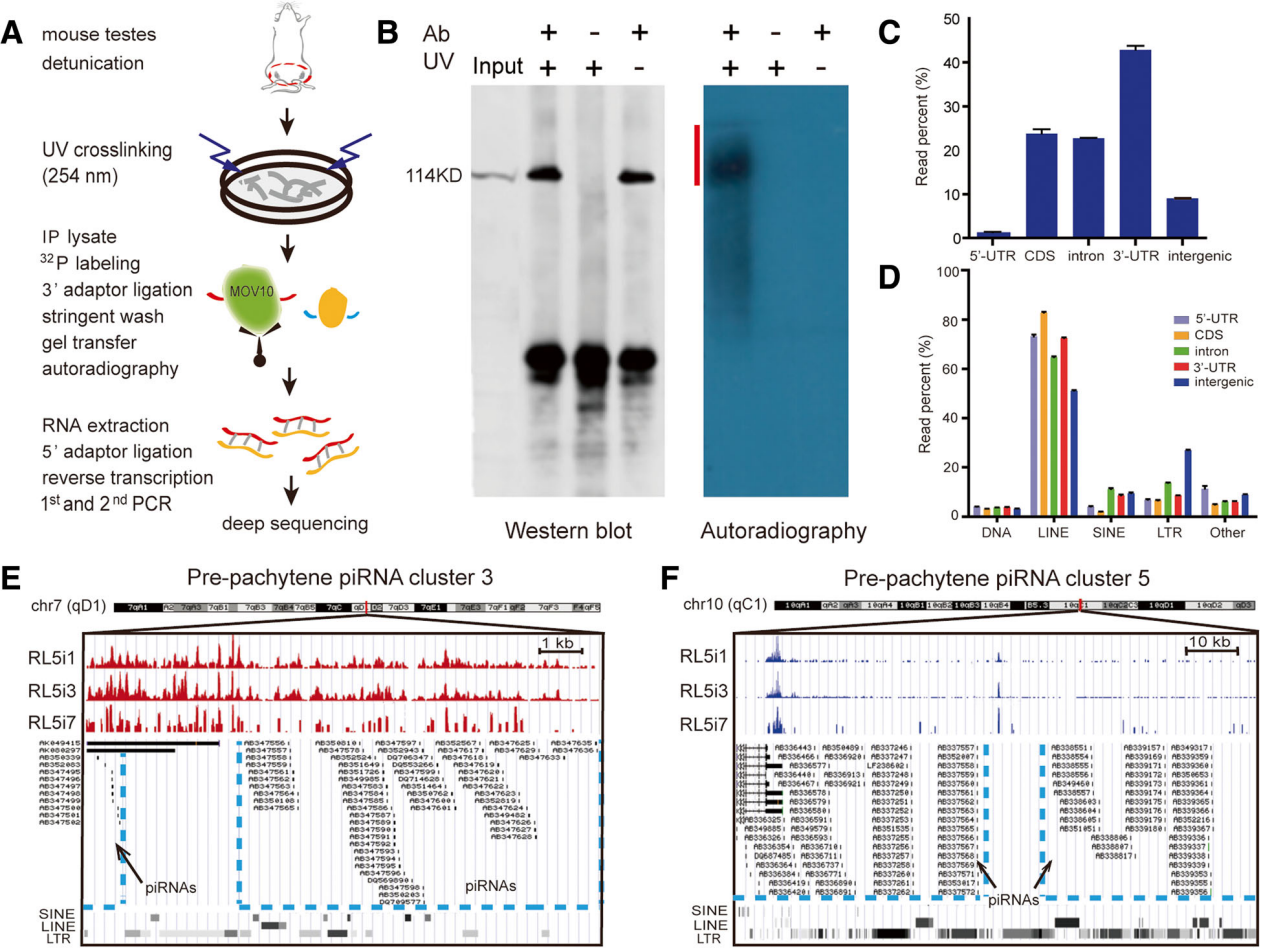
Genomic mapping of in vivo captured MOV10 CLIP targetome. **a** Schematic of the HITS-CLIP procedure. HITS-CLIP employs the following steps to achieve target specificity: covalent crosslinking via ultraviolet irradiation (254 nm), disruption of protein-protein association by highly stringent wash, gel separation of protein-RNA ribonucleoproteins (RNPs) followed by membrane transfer, RNA retrieval, and expansion for deep sequencing. **b** Western blot and autoradiography of MOV10-RNA CLIP complexes. Non-crosslinked testes and IgG CLIP served as negative controls. Three independent libraries were prepared from RNA extracted from gel purified RNPs (marked with red line). **c** Percentage of CLIP reads mapping to genic and intergenic regions. **d** Percentage of CLIP reads mapping to genomic repeat sequences. The error bars in panel **c** and **d** represent variation among three independent CLIP libraries. **e**, **f** Genome browser view of CLIP reads over the genomic window for pre-pachytene piRNA cluster 3 (**e**) and cluster 5 (**f**). The main peak on the left of the piRNA cluster 5 likely reflects 3′-UTR of upstream mRNAs

**Fig. 6:**
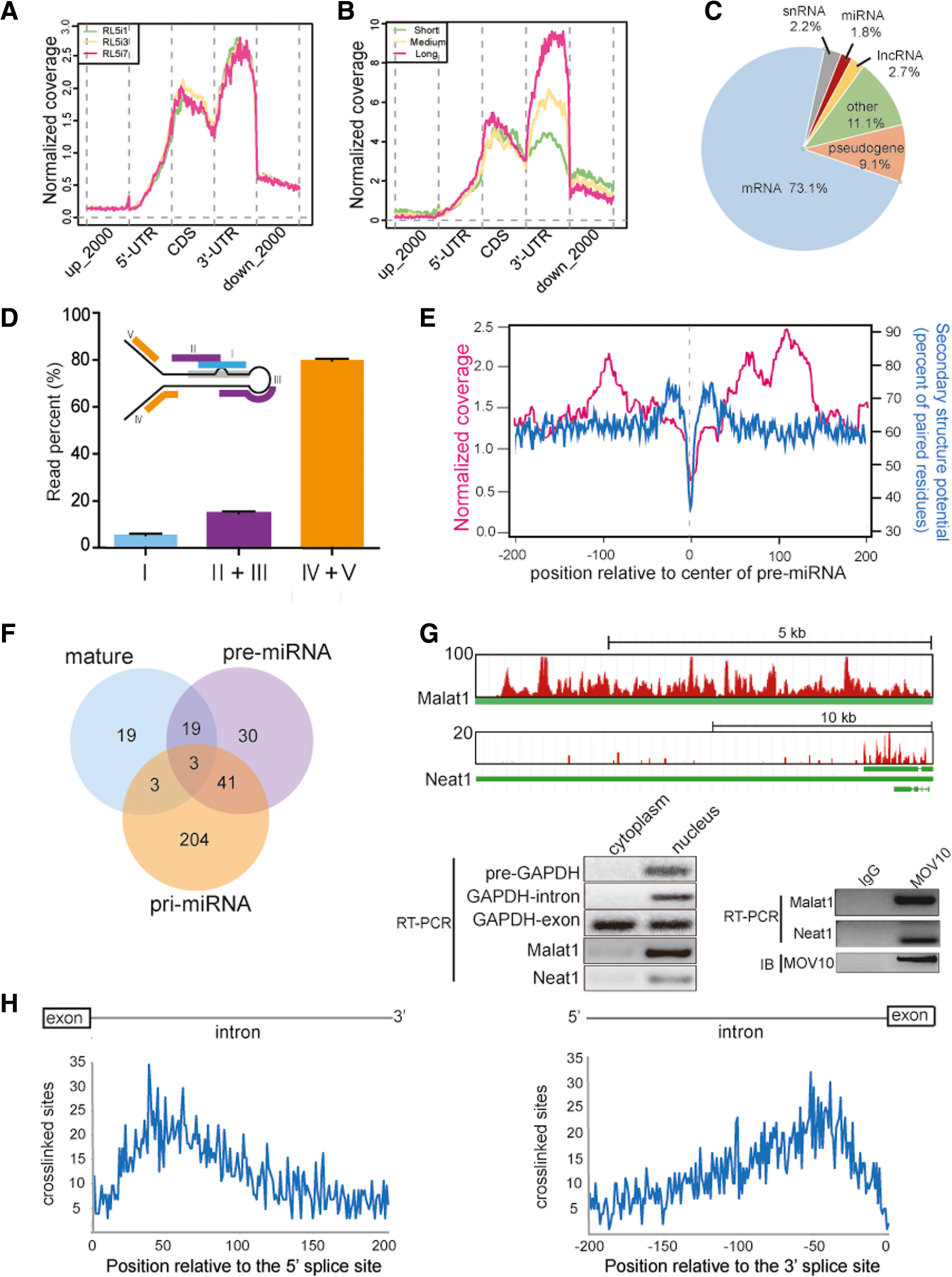
Transcriptome-wide annotation and analyses of MOV10 target RNAs. **a** Distribution of MOV10 CLIP reads mapping to different regions of mRNAs. Three curves represent three CLIP libraries. **b** Distribution of MOV10 CLIP tags mapping to mRNAs with different 3′-UTR length. mRNAs were ranked by 3′-UTR length and divided into terciles containing equal transcript numbers. **c** The pie chart shows the distribution of RNA types identified as MOV10 CLIP targets. **d** Classification of CLIP reads into 5 categories (I–V) based on their relative position to pre-miRNA hairpins. Read distribution of categories I, II+III, and IV+V is calculated. **e** Normalized coverage of MOV10 CLIP reads (red curve) and secondary structure potential of corresponding genomic sequences (blue curve) around pre-miRNAs. MOV10 CLIP tags mapping to ± 200 nt windows flanking the middle point of pre-miRNAs are plotted as density values at single-nucleotide resolution. **f** Numbers of mature miRNA, pre-miRNA, and pri-miRNA bound by MOV10. Overlapping regions represent the miRNAs that are defined as no less than two forms. **g** Genome browser view and validation of MOV10-bound nuclear lncRNAs. UCSC visualization of MOV10 CLIP reads on the lncRNAs *Malat1* and *Neat1*. The green bars represent three isoforms of *Neat1*. RT-PCR confirms nuclear localization of these lncRNAs in P10 testis. The amplicons of pre-GAPDH and GAPDH-intron serve as controls for nuclear fraction and GAPDH-exon serves as control for cytoplasmic fraction. The interaction between MOV10 and lncRNAs was validated by RIP-PCR. **h** MOV10-crosslinked positions within introns. The distribution of genomic crosslinked sites within intronic regions upstream and downstream of the splice site is shown. A genomic crosslinked site was defined as the corresponding genomic position of a deletion identified in a CLIP tag. Each unique position is only represented once

To capture MOV10 footprints in vivo, we chose P10 testis, in which MOV10 is abundant in spermatogonia, and employed HITS-CLIP, a technique enabling transcriptome-wide analysis of protein-RNA interactions (Fig. 5a). The anti-MOV10 antibody used in this study conforms to ENCODE guidelines advised for RNA immunoprecipitation (RIP)-seq (Additional file 1: Figure S1 and Methods: MOV10 Antibody Validation). Specific IP enrichment of MOV10-RNA RNPs was confirmed by western blot analysis and autoradiography, respectively (Fig. 5b). We generated 3 biological replicate CLIP-seq libraries (RL5i1, RL5i3, and RL5i7) with mapping ratios of ~ 85% (Additional file 1: Figure S13) and high reproducibility (Additional file 1: Figure S13). The majority (~ 90%) of MOV10 CLIP tags mapped to genic regions (5′-UTR, CDS, introns, and 3′-UTR), and ~ 10% mapped to intergenic regions (Fig. 5c), contrasting with previous findings with the homolog MOV10L1, for which CLIP tags mostly mapped within intergenic piRNA clusters [38]. Approximately 50% of MOV10 CLIP tags within genic regions and ~ 75% within intergenic regions overlapped with repeat sequences (Additional file 1: Figure S13), which were predominantly (50~80%) long interspersed nuclear element-1 sequences (LINE1) and also included long terminal repeats (LTR) and short interspersed nuclear elements (SINE) sequences (Fig. 5d and Additional file 1: Figure S13). MOV10 CLIP tags overlapped with a subset of the 19 most highly expressed pre-pachytene piRNA clusters [65, 66] (Additional file 1: Table S1), with uniform distribution across piRNA cluster 3, overlapping with retrotransposon and/or piRNA elements (Fig. 5e), but only few dispersed reads on cluster 5 (Fig. 5f). Consistent with observations in cell lines [44], testis-derived MOV10 CLIP tags mapping to genic regions (13,210 mRNAs with FPKM ≥ 0.5 and reproducibility in no less than two CLIP libraries [3]) exhibited a strong enrichment within mRNA 3′-UTRs (Fig. 6a), including the 3′-UTRs of three genes essential in SPCs, *Etv5*, *Bcl6b*, and *Zbtb16* (Additional file 1: Figure S14). Grouping of these mRNAs into terciles according to 3′-UTR length (long, medium, and short 3′-UTR; Additional file 2: Table S2) revealed preferential binding of MOV10 to long 3′-UTRs (Fig. 6b and Additional file 1: Figure S14). Protein-RNA UV crosslinking at binding sites may induce deletions that are caused by errors during reverse transcription, such that deletion analysis can identify local sequence-binding preference [67, 68]. We identified total 33,468 genomic sites with deletions in 297,083 CLIP-seq reads. Crosslinked sites were predominantly located in GC-rich regions (Additional file 1: Figure S14), consistent with a previous report [48]. The areas immediately flanking (~ 5 nucleotides) MOV10 binding sites were AU rich (~ 54%) and had lower GC (~ 46%) content, a feature that was present independently of mRNA 3′-UTR length.

### MOV10 binds to nuclear ncRNA species linked to splicing

Considering the large number of MOV10 CLIP tags mapping to intronic and intergenic regions (Fig. 5c), we asked whether MOV10 extensively binds ncRNAs. To confirm this, we first mapped MOV10 CLIP reads to genome-wide annotations available from Ensemble (Additional file 1: Table S3). Applying the same selection criteria used for mRNAs (FPKM ≥ 0.5, reproducible in two replicates), we found that 26.9% of annotations associated with ncRNAs, including lncRNAs and miRNAs (Fig. 6c). The miRNA-annotated CLIP reads predominantly mapped to miRNA precursors (Additional file 3: Table S4). Because miRBase does not include pri-miRNA coordinates, we classified MOV10 HITS-CLIP reads into five categories (I–V) based on their relative position to the pre-mRNA hairpin (Fig. 6d). Category I mapped to the mature miRNA sequence, category II and III were within the hairpin region and thus putative pre-miRNAs, and category IV and V reads were within a ± 100 nt window of the hairpin region and thus presumptive pri-miRNAs. Most reads mapped to categories IV and V, implying interaction with primary miRNAs transcripts (Fig. 6d). We next analyzed the distribution of CLIP reads relative to the central hairpin sequence of pre-miRNAs and detected enrichment in distal regions (Fig. 6e), predicting that MOV10 may not associate with the classical Microprocessor. MOV10 associated with the following miRNA species: 44 mature miRNAs, 93 pre-miRNAs, and 251 pri-miRNAs (Fig. 6f and Additional file 4: Table S5). LncRNAs encompassed 2.7% of MOV10-CLIP tag annotations and included *Malat1 (Neat2)* and *Neat1* (Fig. 6g)*,* which are known structural components of nuclear (or splicing) speckles and paraspeckles [69–72]. These nuclear subdomains store splicing factors and/or RNA processing factors and are involved in gene expression control. RT-PCR analysis of subcellular RNA fractions confirmed the presence of these nuclear lncRNAs in testis, with *Malat1* being more abundant than *Neat1* (Fig. 6g, left). Association of MOV10 with *Malat1* and *Neat1* RNA in testis was validated by RIP-PCR (Fig. 6g, right). In addition, MOV10-CLIP tags mapped to snRNAs (Fig. 6c), a class of small spliceosomal RNAs found within the nuclear speckles. Splicing regulatory proteins primarily bind to mRNA precursors and affect spliceosome assembly typically at nearby splice site [73]. Intriguingly, we observed enrichment of crosslinked sites/nucleotides at approximately 50 nt upstream of the 3′ splice site or 50 nt downstream of the 5′ splice site (Fig. 6h and Additional file 1: Figure S15). Similarly, short distances between splice site and splice regulator binding sites have been reported elsewhere [74, 75]. To investigate potential causes for preferential binding of MOV10 to these regions, we calculated nucleotide base compositions within ± 100 nt flanking either splice site with crosslinked sites identified on its intronic side. We found that exonic regions exhibited uniform distribution of the four bases (ATCG), whereas intronic regions had obviously higher AT content than CG base content (Additional file 1: Figure S15). Next, we analyzed the base content throughout the intronic regions with splice sites and found that the levels of AT enrichment were relatively low (Additional file 1: Figure S15). However, we did not identify a significant GC enrichment indicative of high secondary structure potential in proximity to MOV10 binding sites, as observed for mRNAs (Additional file 1: Figure S14). Thus, the recruitment of MOV10 to intronic binding sites may involve its interaction with the spliceosome residing near splice sites rather than sequence/structural binding preferences.

### Nuclear MOV10 associates with protein components involved in splicing

**Fig. 7:**
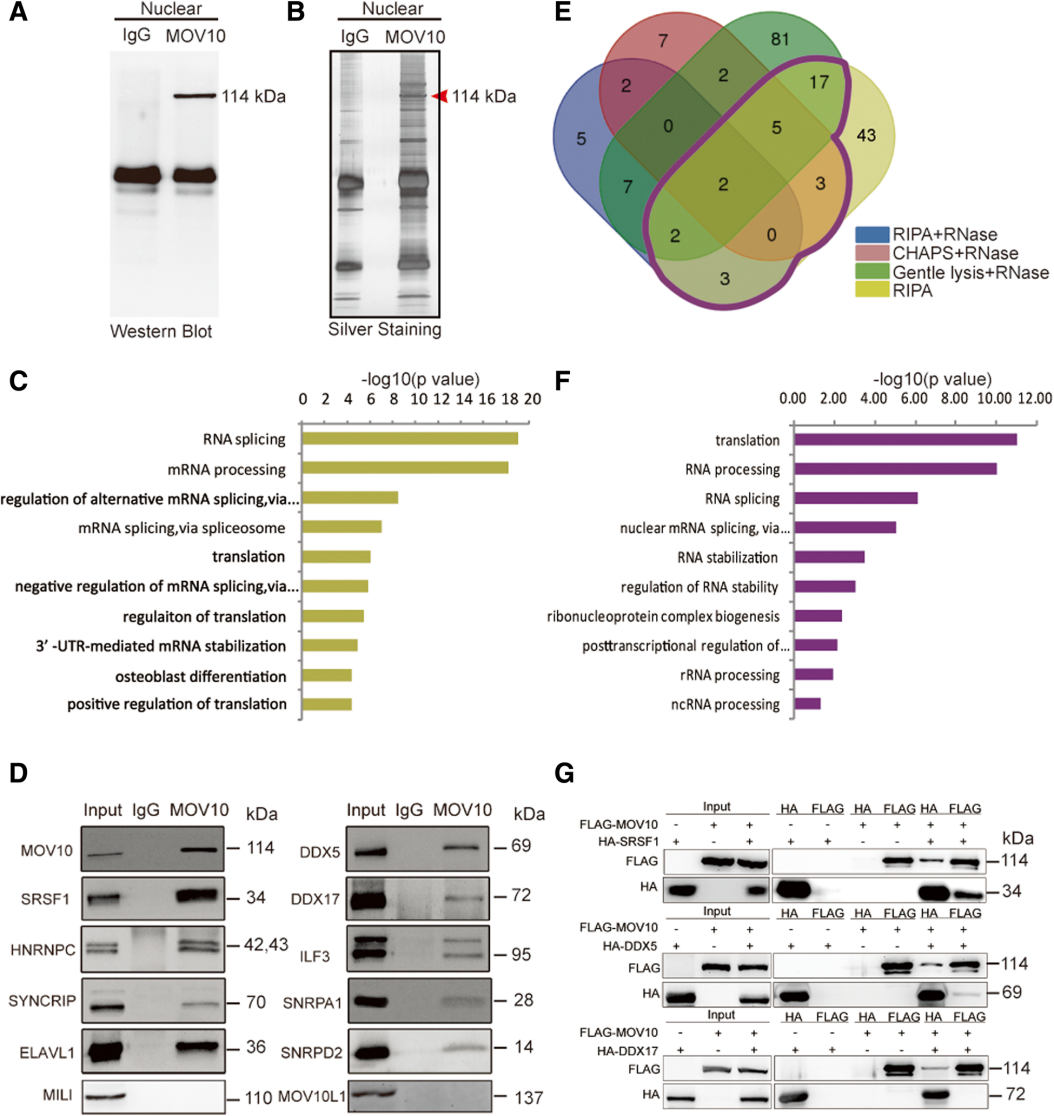
Identification of MOV10-interacting proteins in the nucleus. Western blot (**a**) and silver staining (**b**) of the MOV10 IP complex from nuclear lysate. **c** Gene ontology analysis of MOV10-associated proteins in the nucleus. **d** Nine MOV10-associated splicing-related proteins were selected for validation by IP-western blot assay. The piRNA pathway proteins MILI and MOV10L1 served as negative controls. **e** Venn diagram showing the cross analysis of four sets of MOV10 nuclear IP-MS data. The purple line marks 32 proteins that were reproducibly identified in IP using RIPA buffer without RNase treatment and at least one other IP with RNase treatment. **f** Gene ontology analysis of the 32 reproducible MOV10-assoicated proteins. **g** FLAG-MOV10 and HA-target (SRSF1, DDX5 or DDX17) were overexpressed in HEK293T cells, and IPs were performed by FLAG and HA antibodies, followed by western blot analysis

To identify MOV10-associated proteins, we performed endogenous immunoprecipitation (IP) using nuclear and cytoplasmic fractions of P10 testis. Western blot analyses showed a single prominent band of the predicted size of MOV10 (Fig. 7a, Additional file 1: Figure S16). Mass spectrometry (IP-MS) analysis of nuclear MOV10 IP and IgG control (Fig. 7b) identified 75 candidate proteins with IBAQ intensity enriched more than 10 times, of which 69 proteins were detected only by MOV10 IP (Additional file 5: Table S6). GO term enrichment analysis identified strong associations with RNA splicing followed by RNA processing (Fig. 7c). Consistent with distinct roles of MOV10 in the nucleus and cytoplasm, nuclear and cytoplasmic interaction partners of MOV10 were largely unique to each compartment (Additional file 1: Figure S16 and Additional file 5: Table S6). Nuclear MOV10-associated proteins included 5 proteins (SRSF1, HNRNPC, SYNCRIP, SNRPA1, and SNRPD2) related to the core splicing machinery [23]; 3 RNA processing factors (PABPC1, UPF1, and ELAVL1), which bind to 3′-UTRs and utilize alternative cleavage to regulate mRNA stability and translational efficiency [76]; 2 proteins (DDX5 and DDX17) involved in both splicing and miRNA processing [26, 77]. The Microprocessor components DGCR8 and DROSHA were not detected. IP-western blot analysis validated 9 nuclear MOV10-associated proteins (Fig. 7d). To further characterize these MOV10 interaction partners, we performed additional IP-MS experiments using 3 different RNase-containing lysis buffers of differential stringency and thus capacity to disrupt protein-protein interactions. We identified 32 proteins that were identified both in the original IP-MS and in at least one RNase-treated IP-MS (Fig. 7e and Additional file 5: Table S6), suggesting that these candidates may interact with MOV10 directly. The enriched GO terms for the 32 proteins were related to RNA splicing and processing (Fig. 7f), and among the set were 3 splicing factors (SRSR1, DDX5, and DDX17). Additional co-IP experiments using HEK293T cells overexpressing FLAG-tagged MOV10 and HA-tagged target proteins confirmed that each of the 3 splicing factors SRSR1, DDX5, or DDX17 co-precipitated MOV10, and reciprocally, SRSF1 and DDX5 were co-precipitated by MOV10 (Fig. 7g). In particular, SRSF1 exhibited stronger affinity to MOV10 (Fig. 7 g), consistent with the results from endogenous IP (Fig. 7d). Thus, MOV10 appears to interact with components of the splicing machinery, including SRSF1.

## Discussion

Here, we have investigated the function of the RNA helicase MOV10 in the mammalian male germline. In contrast to its homolog MOV10L1, the role of MOV10 in germ cells has been poorly characterized and understood. We find that MOV10 is abundantly expressed in spermatogonia, is present in both the cytoplasm and the nucleus, and interacts with distinct sets of protein partners in these cellular compartments. Our data reveal that nuclear MOV10 promotes miRNA generation and regulates splicing and that these two processes crosstalk.

Interactions between RNA and RBPs are intricate and involve RNA-binding and other regulatory domains that accommodate the formation of miscellaneous RNPs. MOV10 and MOV10L1 contain a conserved helicase domain but have limited overall consensus sequence similarity (Additional file 1: Figure S3). MOV10 associates with polysomes whereas MOV10L does not (Additional file 1: Figure S3). MOV10L1 is restricted to the testis and engages in the piRNA biogenesis pathway by direct interaction with Piwi proteins [35, 38]. In contrast, MOV10-bound repeat elements are not bona-fide pre-pachytene piRNA precursors. They do, however, exhibit some overlap because pre-pachytene clusters are enriched for repeat sequences, but there is no direct evidence linking MOV10 to the piRNA pathway. MOV10 is expressed ubiquitously across all tissues and has been shown to contribute to a variety of cytoplasmic functions [42, 44, 45, 53, 78, 79]. MOV10 associates with the AGO proteins of the RNA-induced silencing complex (RISC) and is required for miRNA-guided gene silencing [47, 49, 50, 80, 81]. MiRNA-guided translational regulation is mediated by the interaction of MOV10 with the RISC-associated factor Fragile X Mental Retardation Protein (FMRP) [48, 82, 83]. Whereas MOV10L1 is a known cytoplasmic nuage component, MOV10 localizes to both the cytoplasm and nucleus of male germ cells suggesting cytoplasm-to-nucleus shuttling in early postnatal testis (Fig. 1 and Additional file 1: Figure S1), similarly to observations as reported for early postnatal neurons in the brain [53]. The largely mutually exclusive expression patterns of MOV10 and MOV10L1 in spermatogenesis suggest that these helicases have non-overlapping roles during distinct developmental phases of germ cell maturation.

In the present study, we find that MOV10 is a transcriptome-wide RNA regulator in germ cells, affecting critical protein-coding genes (*Etv5*, *Bcl6b*, and *Zbtb16*) and miRNAs (miR21a, miR20a and miR106a) that are involved in SPC proliferation and/or self-renewal. These findings identify MOV10 as a novel determinant for SPC fate, confirmed by functional studies demonstrating that MOV10 deficiency impairs proliferation and repopulation capacity of SPCs. We also show that MOV10 targets pri-miRNAs (Fig. 6d–f), which are processed in the nucleus. Knockdown of *Mov10* resulted in a global decrease of miRNAs (Fig. 3a–c). The miRNA pathway initiates with transcription of primary miRNA transcripts. Our results show that the reduction in miRNA levels is associated with a reduction in miRNA transcript levels, as shown for miR21a and miR20a, implying MOV10 in miRNA processing (Fig. 3d–h). We isolated nuclear MOV10 complexes and identified components involved in miRNA processing (Fig. 7). MOV10 also interacted with two nucleus-specific lncRNAs in germ cells, *Malat1* and *Neat1.* These are well-defined structural lncRNAs for building and/or maintaining nuclear bodies that are involved in activities of RNA splicing and/or processing [69–72]*.* A recent study reported that *Neat1* scaffolds RBPs and the Microprocessor to globally enhance pri-miRNA processing [84]. Our findings point to a completely novel role of nuclear MOV10 in boosting miRNA production.

It has become increasingly evident that the level of a mature miRNA is determined by both the transcription rate and the splicing/processing efficiency of the miRNA precursor, and these processes are governed by a large spectrum of RBPs [85]. Multitask functionality of RNA helicases underpins their importance in remodeling splicing complexes by dynamic recruitment of splicing factors prior to nuclear export [26]. Splicing, constitutive or alternative, occurs co-transcriptionally and impacts profoundly on post-transcriptional gene regulation [24, 86], miRNA biogenesis [28, 31, 87], 3′-UTR maturation [76, 88], lncRNA processing [25], among others. The presence of a long 3′-UTR has been shown to trigger nonsense-mediated mRNA decay (NMD) by recruiting NMD factors such as UPF1 [89] and MOV10 coordinates with UPF1 in 3′-UTR-mediated NMD [44]. Our results showing preferential binding of MOV10 to long 3′-UTRs (Fig. 6b), combined with functional data showing that transcripts with long 3′-UTRs preferentially accumulate in the absence of MOV10 (Additional file 1: Figure S6), suggest that MOV10 may play a role in processing long 3′-UTRs. Interestingly, we found that decrease of the miR21a precursor level was concurrent with miR21a retention in an alternative 3′-UTR event (Fig. 3i). Alternative polyadenylation of *Vmp1* gene transcripts has been proposed to regulate the expression of miR21a [63]. Mirtron is an intriguing class of intronic miRNA identified through experimental and/or computational methods and processed via a non-canonical miRNA pathway that is mediated by alternative splicing [30, 64]. Such alternative splicing events may also be the prerequisite for the production of miRNAs from other intronic miRNA precursors. Retention of these intronic miRNAs attenuates miRNA precursor processing. In this study, we demonstrate that the MOV10 serves as a splicing regulator for mirtron in SPCs. Many mirtron retention events are concurrent with decrease of the mirtron transcript levels (Fig. 4 and Additional file 1: Figure S10), suggesting crosstalk. We found that MOV10 intronic binding sites tend to reside in proximity to splice sites (Fig. 6h and Additional file 1: Figure S15). A recent transcriptome-wide analysis discloses that intronic AU-rich element (ARE) is more abundant than that of 3′-UTR [90]. MOV10-bound intronic sequences lack a bias of GC-binding but are more enriched in AU than the average across the whole genome (Additional file 1: Figure S15). MOV10 is the vertebrate homolog of Drosophila Armitage, which is essential for both RNA interfering and piRNA pathways [91, 92]. Sequence analysis revealed the identity between MOV10 and Armitage is only 23.21%, but the identity between mouse and human MOV10 reaches 91.4%. In human cell lines, MOV10 is implicated in regulating splicing, evidenced as one of the identified candidates within the functional splicing regulatory network [23]. These data, along with MOV10 interaction with splicing factors (Fig. 7), support a direct involvement of MOV10 in splicing regulation. Among the MOV10 protein partners, SRSF1 is a prototypical splicing factor protein that, in addition to its function in splicing, also plays a role in mRNA export, NMD, and translation [93, 94].

**Fig. 8:**
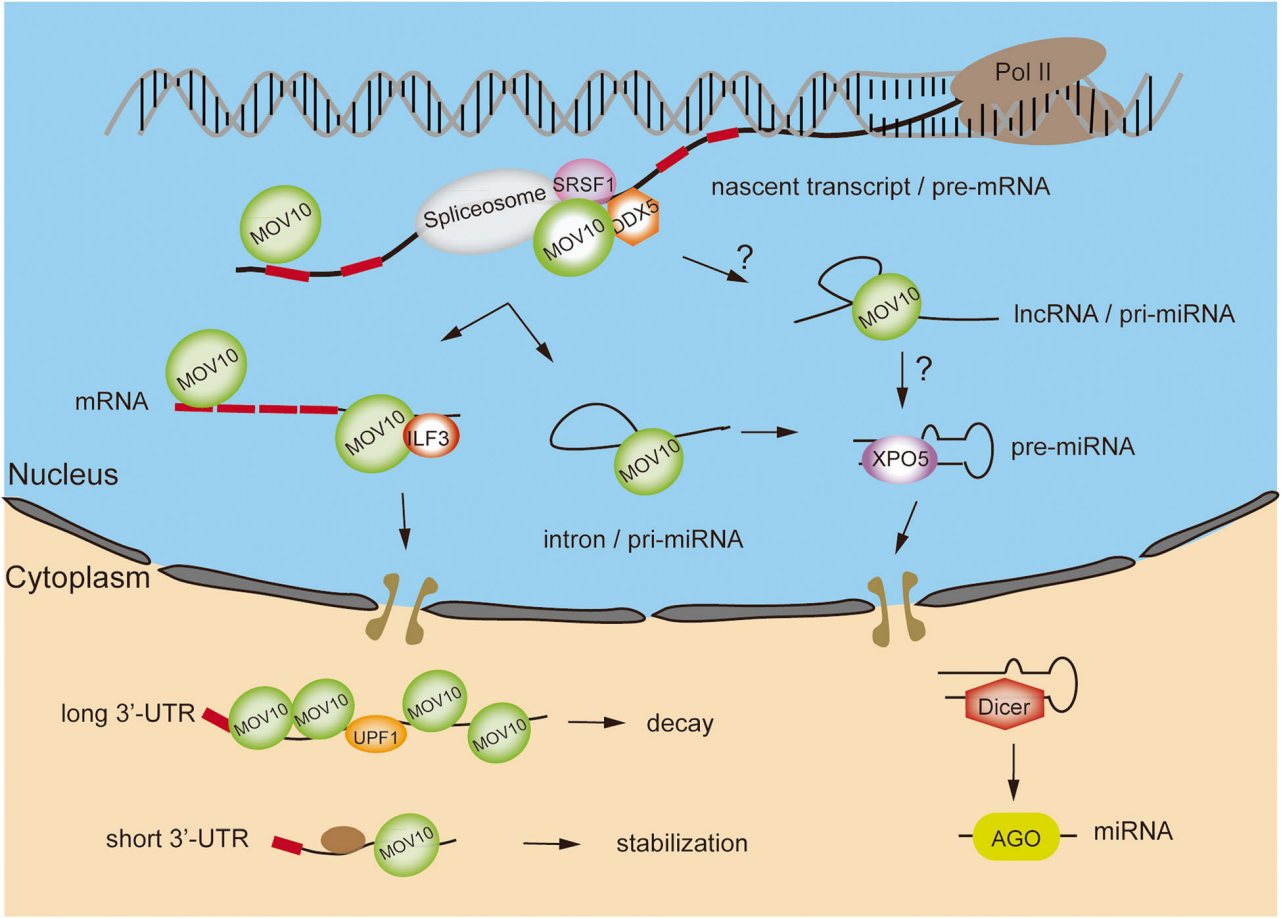
Subcellular function of MOV10 in germ cells. Diagram of proposed RNA regulatory mechanisms executed by MOV10 in interaction with diverse RNA species and multiple proteins. MOV10-mediated RNA regulation pathways are initiated in the nucleus, where MOV10 associates with splicing factors, such as SRSF1 and DDX5, to bind intronic regions of pre-mRNA. The splicing process is mechanistically coupled with transcription and RNA processing, linking directly or indirectly to the fate of nascent transcripts for miRNA, lncRNA, mRNA, or even 3′-UTR. In the case of *Mov10* deficiency, this coordinated regulation is severely impaired, globally impairing RNA splicing/processing activity, and thus failure to maintain a normal balance of various RNA pools, for example, as illustrated here, MOV10-mediated splicing regulation of intronic miRNAs. MOV10 transports its target RNAs from the nucleus to the cytoplasm to carry out further maturation, degradation, or translation. MOV10 predominantly binds long 3′-UTR as a mediator of mRNA decay via interaction with the NMD factor UPF1. Thus, the disruption of MOV10-mediated RNA homeostasis could reflect a profound, combinatorial effect resulting from a cascade of hierarchical events causing RNA dysregulation

In summary, our study has established previously undescribed links between nuclear MOV10, miRNA biogenesis, and splicing in mammalian germ cells. Integrated with other findings, we propose a model in which the role of MOV10 in orchestrating multiple regulatory RNA species begins in the nucleus (Fig. 8). We hypothesize that MOV10 acts like a scaffold that governs both protein-protein and protein-RNA interaction networks. These studies may be deepened or extended through further characterization of MOV10 CLIP targets exclusively from the nuclear fraction, which would augment the read coverage on MOV10-bound primary transcripts, and use of a robust cell line given that such MOV10 nuclear function is conserved. Our study sheds new light on the intracellular pathways from inside nucleus to cytoplasm and provides a framework for how MOV10 controls RNA fates at multiple levels.

## Conclusions

In this work, we employed multiple methodological approaches to study the role of MOV10 in mammalian germ cells and made several discoveries. First, MOV10 is a nucleocytoplasmic protein that is mainly expressed in spermatogonia. Second, MOV10 shapes the transcriptome and determines the fate of spermatogonial progenitor cells (SPCs). Third, MOV10 regulates miRNA biogenesis via nuclear RNA metabolism and splicing control. Lastly, MOV10 binds to nuclear RNA elements and interacts with multiple nuclear splicing factors including SRSF1. These findings provide new knowledge of the mechanisms underlying the involvement of this versatile RNA helicase in a broader RNA regulation network.

## Materials and methods

### Mice, plasmids, and antibodies

C57BL/6 and 129/SvCP mice were obtained from the Model Animal Research Center of Nanjing University. C57BL/6 ×129S-Gt (Rosa) 26Sor/J mice were obtained from the Jackson Laboratory. All mice were housed with 12/12-h light/dark cycles, at 22 °C, and allowed free access to water and food.

The pLKO.1 shRNA vector and lentivirus packaging plasmids (pmd-REV and pmd-1G/pmd-LG) were provided by Dr. Xin Wu (State Key Laboratory of Reproductive Medicine, Nanjing Medical University, China). Two pLKO.1 shRNA constructs (shMov10-832 and shMov10-833) were designed with shRNA sequences shown in Additional file 6: Table S7. To construct the pCDH-MOV10 plasmid, *Mov10* full length CDS cloned from mouse testis was inserted into the pCDH-EF1-MCS-T2A-puro plasmid using EcoRI/BamHI restriction sites. Plasmids for the expression of FLAG-MOV10 (ENSMUSG00000002227,1004 aa), HA-SRSF1 (ENSMUSG00000018379, 248 aa), HA-DDX5 (ENSMUSG00000020719, 615 aa), and HA-DDX17 (ENSMUSG00000055065, 652 aa) were constructed by cloning *Mov10*, *Srsf1*, *Ddx5*, *and Ddx17* full length CDS into the pRK5 vector with either FLAG or HA tag at their N terminus.

Primary antibodies used in this study were rabbit anti-MOV10 (10370-1-AP, Proteintech, RRID:AB_2297897), rabbit anti-MOV10L1 (UP2175, [35]), rabbit anti-ETV5 (13011-1-AP, Proteintech, RRID:AB_2278092), rabbit anti-BCL6b (DF9075, Affinity), goat anti-PLZF/ZBTB16 (AF2944, R&D, RRID:AB_2218943), rabbit anti-LIN28 (ab46020, Abcam, RRID:AB_776033), mouse anti-β-Actin (A5316, Sigma, RRID:AB_476743), rabbit anti-β-Tubulin (ab6046, Abcam, RRID:AB_2210370), mouse anti-GAPDH (MB001, Bioworld Tech), goat anti-RPL22 (NBP1–06069, Novus Biologicals, RRID:AB_2181599), mouse anti-Histone H3 (05-499, Millipore, RRID:AB_309763), rabbit anti-ELAVL1 (ab200342, Abcam, RRID:AB_2784506), rabbit anti-ILF3 (ab92355, Abcam, RRID:AB_2049804), rabbit anti-DHX9 (ab26271, Abcam, RRID:AB_777725), rabbit anti-DDX17 (ab70184, Abcam, RRID:AB_1209629), mouse anti-SRSF1(32-4500, Thermo, RRID:AB_2533079), rabbit anti-SNRPA1(ab128937, Abcam, RRID:AB_11139816), rabbit anti-SNRPD2 (ab198296, Abcam), rabbit anti-HNRNPC (ab133607, Abcam), rabbit anti-DDX5 (ab21696, Abcam, RRID:AB_446484), mouse anti-SYNCRIP (ab184946, Abcam), rabbit anti-MVH (ab13840, Abcam, RRID:AB_443012), rabbit anti-MILI (ab36764, Abcam, RRID:AB_777284), rabbit anti-DROSHA (55001-1-AP, Proteintech, RRID:AB_10859254), rabbit anti-DGCR8 (10996-1-AP, Proteintech, RRID:AB_2090987), rabbit anti-DICER (A6021, ABclonal, RRID:AB_2766716), and normal rabbit IgG.

### MOV10 antibody validation

In alignment with ENCODE guidelines (version 2.0, 9 January 2012), the rabbit anti-MOV10 (10370-1-AP, Proteintech) which was used for all experiments in this study was validated as follows. Outcomes of western blot analyses were consistent with results obtained with a second commercially available rabbit anti-MOV10 antibody (ab80613, Abcam); both antibodies identified the same single band in MOV10 IP complexes from testis produced using 10370-1-AP (Additional file 1: Figure S1), and in lysates from FLAG-MOV10 overexpressing HEK293T cells (Additional file 1: Figure S1); both 10370-1-AP (Proteintech) and anti-FLAG antibodies identified the same band in FLAG IP complexes from HEK293T cells (Additional file 1: Figure S1). Crosslinking IP (CLIP)-WB (Fig. 5b) and IP-MS (Fig. 7a) using 10370-1-AP (Proteintech) identified a single strong band of the predicted size, and among cytoplasmic proteins, MOV10 had the largest number of unique peptides identified and highest iBAQ value, confirming high affinity and specificity of the antibody used for IP (Additional file 5: Table S6). No cross reactivity with similar proteins was observed when anti-MOV10L1 was used for MOV10 IP complex (Fig. 7d). MOV10 immunostaining of cryosections from E16.5, P1, P10 testes, and SPCs and purified Spg from P6–8 testes produced a signal (Fig. 1e–g and Additional file 1: Figure S1) consistent with nuclear IP and WB results (Fig. 7a).

### Purification of spermatogenic cells

We isolated three representative types of germ cells, spermatogonia (Spg), pachytene spermatocyte (PS), and round spermatid (RS), using the STA-PUT method of sedimentation velocity at unit gravity at small scale [95]. Seminiferous tubules were isolated from decapsulated testes from 2-month-old adult (for PS and RS) or P6-P8 mice (for Spg) by incubation in DMEM with collagenase (1 mg/ml) at 37 °C for 15 min with shaking. Tubules were digested in Trypsin (0.25%) and DNase (1 mg/ml) in DMEM at 37 °C for 15 min and then filtered to obtain single cell suspensions. Germ cell populations were separated using a BSA gradient using the STA-PUT Velocity Sedimentation Cell Separator (ProScience Inc. Canada). Sequential fractions were collected, and cell types determined based on morphology. Based on morphologic characteristics and cell diameter, cell purities of isolated stage-specific germ cell populations were determined. The purity of isolated Spg, PS, and RS was approximately 80%, 80%, and 90%, respectively. Cell fractions of uniform populations were pooled, pelleted, and stored for subsequent analyses.

### Immunofluorescence microscopy

To prepare frozen sections, testes were fixed in 4% paraformaldehyde (PFA) at 4 °C overnight, embedded and sectioned (6 μm). The sections were treated with 6 μM DTT and 10% serum in TBST (1 × TBS containing 0.1% Tween 20) for 1 h at room temperature (RT), then incubated overnight at 4 °C with primary antibodies diluted in 10% serum in TBST. After incubation with secondary antibodies, the sections were then washed in TBS three times and stained with DAPI (Vector Laboratories). For whole-mount assay, seminiferous tubules from adult testis were prepared as previously described with modifications [54]. Testis tubules were digested with trypsin and collagenase, washed with PBS, and fixed with 4% PFA for 2 h. After blocking with 10% serum in TBST (1 × TBS containing 0.1% Tween 20) for 1 h at RT, the samples were incubated overnight at 4 °C with anti-MOV10 antibody (1:25) and anti-PLZF antibodies (1:200), washed three times in TBS, and incubated with Texas red or FITC-conjugated secondary antibodies (Jackson Immuno Research) for 1 h at RT. Samples were washed as before and mounted in microslide shield with DAPI. Immunofluorescence for all samples was examined under laser scanning confocal microscope (Carl Zeiss, LSM700).

### SPC cell culture, transduction, and transplantation

Establishment of long-term SPC cultures has been described previously [20]. For cell culture, we isolated Thy1-positive cells from 6- to 8-day-old B6;129S-Gt (Rosa) 26Sor/J mice and cultured these on 12-well plates with mitotically inactivated STO (SIM mouse embryo-derived thioguanine and ouabain-resistant feeder, SNLP76/7-4, ATCC) feeder layers in a defined serum-free, consisting of minimal essential medium (MEMa, Life Technology) supplemented with 2% bovine serum albumin (BSA, Sigma-Aldrich, St. Louis, MO, USA), 20 ng/ml GDNF (R&D Systems, Minneapolis, MN, USA), 150 ng/ml GFRA1 (R&D Systems) and 1 ng/ml basic fibroblast growth factor (FGF2; BD Biosciences), 10 μg/ml transferrin (Sigma-Aldrich), 50 μM free fatty acid mixture (5.6 mM linolenic acid, 13.4 mM oleic acid, 2.8 mM palmitoleic acid, 35.6 mM linoleic acid, 31.0 mM palmitic acid, 76.9 mM stearic acid; all from Sigma-Aldrich), 30 nM Na_2_SeO_3_ (Sigma-Aldrich), 2 mM L-glutamine (Life Technology), 50 μM 2-mercaptoethanol (Sigma-Aldrich), 5 μg/ml insulin (Sigma-Aldrich), 10 mM HEPES (Sigma-Aldrich), and 60 μM putrescine (Sigma-Aldrich). Medium was replaced every 2–3 days.

Lentivirus particles were generated following Addgene protocols (http://www.addgene.org/tools/protocols/plko/). The pLKO.1 shRNA plasmids (shVector or shMov10) and lentivirus packaging plasmids (pmd-REV and pmd-1G/pmd-LG) were co-transfected into HEK293T cells, and supernatant containing lentivirus particles was harvested after 48 h transfection. For *Mov10* knockdown, 2.5 × 10^5^ SPCs were plated onto 12-well plates pre-coated with 0.1% gelatin (Sigma-Aldrich), and 12 h later, transduced overnight with an equal mixture of culture medium and lentiviral supernatant, supplemented with 5 μg/ml polybrene. Cells were washed, replated on STO feeder layers, cultured for 72 h, and harvested for RNA and protein isolation.

For transplantation experiments, cell suspensions (transduced by shVector or shMov10) were harvested at 72 h post transduction and live cells were enriched by 30% percoll density gradient centrifugation to remove dead cells and debris. One hundred thousand SPCs (GT-Rosa 26Sor/J) were transplanted into each testis of 129/SvCP ×C57BL/6 F1 male mice, in which endogenous spermatogenesis had been depleted by treatment with busulfan (55 mg/kg; (Sigma-Aldrich) at the age of 8 weeks and 5 weeks prior to transplantation. Two months after transplantation, testes were harvested and donor-cell derived colonies visualized with X-gal staining. All animals used in this study were housed under the 12-h light/12-h dark cycles in a specific pathogen-free barrier facility. All experiments and procedures were approved by the Institutional Animal Care and Use Committee of Nanjing Medical University (ID: IACUC-1601287).

### Vector construction, lentivirus packaging, and testis transduction

Two pairs of cDNA oligonucleotides targeting the mouse *Mov10* mRNA were designed. The off-target effects of both sets of the *Mov10* shRNAs were detected by Sylamer program, and analysis indicated that the seeding sequences of both *Mov10* shRNAs were specifically enriched on *Mov10* gene only, but not on other genes. Then, oligonucleotides were synthesized (see Additional file 6: Table S7) and inserted into pSilencer-H1-LV [57, 58], which carries a CMV-driven EGFP reporter downstream of H1-driven shRNA. shRNA and Flag-Mov10 overexpression vectors were first co-transfected to 293T cells to test its efficacy of gene silencing before lentiviral packaging. If these shRNAs were efficient, pSilencer-shRNA lentiviral vectors and packaging plasmids were co-transfected in 293T cells to produce recombinant lentiviral vectors using the calcium phosphate method. After transfection of 293T cells for 48 h, the viral supernatant was filtered through 0.45-μm cellulose acetate filters and harvested. After that, the viral supernatant was spun at 120,000×*g* for 90 min at 4 °C; then, appropriate PBS was used to resuspend the viral pellet for preparing high-titer lentivirus (> 10^8^ transduction units/ml). After adult mice were anesthetized by tri-bromoethanol, one testis was pulled out from the abdominal cavity. A mixture of 10 μL of fresh high-titer lentivirus and 1 μL trypan blue was injected into seminiferous tubule through the microinjection apparatus (FemtoJet 4i, Eppendorf) under a stereoscopic microscope. The testis was returned to the abdominal cavity; then, the abdominal wall and skin were closed with sutures. Each injected mouse was marked and kept warm until they wake up. Mouse testes were harvested after 1–2 weeks recovery and fixed in 4% paraformaldehyde immediately. After testis was packed by O.C.T. compound, 5-μm cryosections of testis were cut and used for TUNEL assay.

### Flow cytometric studies

Apoptosis was examined using FITC Annexin V Apoptosis Detection Kit I (BD Bioscience). SPCs were harvested 96 h after transfection, washed twice with cold PBS buffer, and resuspended in 1× binding buffer at a final concentration of ~ 1 × 10^6^ cells/ml. Five-microliter FITC Annexin V and 5-μl PI solution were added to 100-μl cell suspension, which was then incubated in the dark for 15 min at RT, followed by addition of 400 μl 1× binding buffer within 1 h. For cell cycle analysis, we used the PI/RNase Staining Buffer (BD Bioscience) followed by fluorescence activated cell sorting (FACS). Per assay, two independent experiments with triplicate samples were performed.

### Preparation of CLIP-seq libraries

For each CLIP replicate, 15 pairs of P10 testes were detunicated, UV-crosslinked, flash-frozen in liquid nitrogen, and then stored as pellet at − 80 °C. When processed for CLIP, pellets were lysed with PMPG buffer, treated with DNase, and then centrifuged. The supernatant from the treated lysates was precleared by rabbit IgG and then immunoprecipitated with ~ 5 μg anti-MOV10 antibody using protein A Dynabeads. Meanwhile, 3′-RNA linkers (RL3) were labeled with ^32^P and ligated to CIP (calf intestinal phosphatase)-treated RNA on beads. After stringent wash steps, crosslinked MOV10 RNPs were eluted from beads with Novex reducing loading buffer, separated by electrophoresis in NuPAGE precast gels (4–12% gradient) with MOPS buffer, and transferred onto nitrocellulose (Invitrogen LC2001). Membranes were exposed to film overnight, and fragments containing the main radioactive signal were excised. Library construction, including RNA extraction, 5′ linker ligation, RT-PCR, second PCR, electrophoretic separation, and extraction were performed as described previously [38, 96]. For deep sequencing, we prepared a multiplexed library consisting of three independent MOV10 HITS-CLIP libraries with identifying 3′ barcodes (RL5i1(AUCACG), RL5i3(UUAGGC), and RL5i7(CAGAUC)).

### Isolation of nuclear and cytoplasmic fractions

Subcellular extracts were prepared as described [97], with minor modifications (see related data in supplemental material). One hundred-milligram P10 testis tissue was homogenized in 1 ml Cytoplasmic Extraction Buffer (250 mM sucrose, 10 mM Tris-HCl (pH 8.0), 10 mM MgCl_2_, 1 mM EGTA, 1× protease inhibitor cocktail III) with 100 strokes. Nuclei were pelleted by centrifugation at 300×*g* for 5 min, and the supernatant was collected as cytoplasmic fraction. The nuclear pellet was washed three times in Cytoplasmic Extraction Buffer, resuspended in Nuclear Extraction Buffer (250 mM sucrose, 10 mM Tris-HCl (pH 8.0), 10 mM MgCl_2_, 1 mM EGTA, 0.1% Triton X-100, 0.25% NP-40, and 1× protease inhibitor cocktail III) with 40 strokes, and centrifuged at 100×*g* for 30 s. The supernatant was collected as nuclear fraction. Fractionation efficiency was validated by western blot using antibodies specific for cytoplasmic (GAPDH) and nuclear (histone H3) proteins.

### Immunoprecipitation and mass spectrometry (IP-MS)

Nucleus and cytoplasm were separated via centrifugation at 300×*g* for 5 min as described above. For nuclear MOV10 IP, the nuclear pellet was washed three times with Cytoplasmic Extraction Buffer and lysed separately with 3 ml different IP buffers with or without 10 μg/ml RNase A/T1 (EN0551, Thermo Fisher), including RIPA buffer (50 mM Tris, 150 mM NaCl, 1% NP-40, 1 mM DTT, 0.5% sodium deoxycholate, 0.05% SDS, 1 mM EDTA, and protease inhibitor cocktail), CHAPS buffer (40 mM Hepes, 120 mM NaCl, 1 mM EDTA, 0.3% CHAPS, 50 mM NaF, 10 mM β-glycerophosphate, 10 mM sodium pyrophosphate, 0.5 mM sodium orthovanadate and protease inhibitor cocktail), and gentle lysis buffer (50 mM Tris, 150 mM NaCl, 1 mM EDTA, 0.5% Triton X-100, 10% Glycerol, 50 mM NaF, 5 mM β-glycerophosphate, and protease inhibitor cocktail). For cytoplasmic IP, RIPA buffer was used with no RNase treatment. The supernatant from nuclear or cytoplasmic lysate was collected for IP via centrifugation at 12000×*g* for 10 min and was then precleared with Protein A Agarose Beads (Millipore) for 30 min at 4 °C with rotation, followed by centrifugation and supernatant collection. Rabbit polyclonal anti-MOV10 antibodies (10 μl) or rabbit IgG (3 μl) were added to the supernatant, followed by overnight incubation at 4 °C with rotation. Beads were added to the tube again and incubated 3 h at 4 °C with rotation. Beads were then washed three times with lysis buffer, resuspended in sample loading buffer, boiled, and separated by SDS-PAGE.

MS analysis of proteins was performed by Proteomics Core Facility of Nanjing Medical University. Briefly, gel slices were dissolved in 0.1% formic acid and filtered through a 0.45-μm membrane. Samples were separated by an Ultimate 3000 nano-LC system (Dionex) by loading onto a trap column, followed by automatic submission to a matrix-assisted laser desorption/ionization time-of-flight/time-of-flight (MALDI TOF/TOF) apparatus (ultrafleXtreme, BrukerDaltonics, Bremen) operated in the positive ion mode. MassLynx™ software (version 4.1, Waters Corporation) was used for information collection and MS analysis.

### Cell line co-expression and co-immunoprecipitation (co-IP)

We co-expressed FLAG-tagged MOV10 and HA-tagged SRSF1, HA-tagged DDX5 or HA-tagged DDX17 in HEK293T cells. After transfection of cells for 48 h, the 10-cm dishes were washed three times with cold PBS and then lysed in 50 mM Tris, 150 mM NaCl, 1 mM EDTA, 0.5% Triton, 10% Glycerol, 50 mM NaF and 5 mM β-glycerophosphate and protease inhibitors cocktail for 10 min on the ice. The total cell lysate was spun at 12,000×*g* for 10 min at 4 °C. After centrifugation, supernatants were pre-cleared with 50 μl sepharose protein A beads. Fifty-microliter protein A beads were washed three times with 20 mM sodium phosphate (pH 7.0) and incubated with anti-FLAG or anti-HA antibodies at 4 °C for 3 h. Then, the pre-cleared lysate was incubated with the antibody coated beads at 4 °C for 3 h. The beads were collected by gentle centrifugation (500×*g* for 1 min at 4 °C) and then washed three times with wash buffer (50 mM Tris-HCl (pH 7.4), 150 mM NaCl, 0.1% Triton X-100, 1 mM EDTA), and incubated at 95 °C for 8 min with SDS loading buffer. IP complexes were analyzed by western blot with anti-FLAG and anti-HA antibodies.

### RNA immunoprecipitation (RIP)

Protein A Agarose Beads were washed three times in NT2 buffer (50 mM Tris-HCl (pH 7.5), 150 mM NaCl, 1 mM MgCl_2_, 0.05% NP40, protease inhibitor cocktail, and RNase inhibitor) and coated with 5 μg MOV10 antibody and Normal rabbit IgG in 500 μl NT2 buffer for 3 h at 4 °C. Fifteen pairs of detunicated testes (P10) were homogenized in lysis buffer (100 mM KCl, 5 mM MgCl_2_, 10 mM HEPES, 0.5% NP-40 containing 10 U/ml RNase inhibitor (Promega) and a protease inhibitor cocktail (Roche)) for 1 h at 4 °C. The lysate was centrifuged at 20,000×*g* for 30 min and the supernatant precleared by incubation with beads for 1 h, followed by incubation with antibody- or IgG-bound beads for 5 h. After stringent washing with NT2 buffer and digestion with protease K, total RNAs were extracted with TRIzol.

### Reverse transcription-quantitative real-time polymerase chain reaction (RT-qPCR)

RNA samples were reverse transcribed using PrimeScript RT reagent Kit (Takara) and random primers. As amplification of individual mature miRNAs is inefficient due to their short length, we used specific stem loop primers that extend the miRNA template for subsequent qPCR detection [98]. qPCR analysis was performed using SYBR Premix Ex Taq II mixture (Takara) and the StepOne plus real-time PCR system (StepOne Plus, Life Technology). *36b4* serves as internal control for transcripts and *U6* for mature miRNAs. Oligonucleotide primer sequences are provided in Additional file 6: Table S7.

### Sucrose gradient polysome fractionation

Approximately 20 P10 testes were harvested for polysome fractionation assays. Testis lysates were prepared in a buffer containing 100 mM KCl, 0.1% Triton X-100, 50 mM HEPES, 2 m MgCl_2_, 10% glycerol, 1 mM DTT, 20 U/ml Protector RNase Inhibitor (Promega) and 1 × EDTA free protease inhibitor cocktail (Roche), and kept on ice for 15 min before centrifugation at 10,000*g* for 10 min. The supernatant was carefully loaded on 20 to 50% *w*/*v* linear density sucrose gradient (Gradient Master, Biocomp, Fredericton, NB, Canada) and centrifuged at 38,000 rpm, for 3 h (Beckman Coulter Optima L-100XP Ultracentrifuge, Brea, CA, USA). RNP, 40S to 80S ribosome, and polysome fractions were collected using a piston gradient fractionator (Biocomp). The efficiency of polysome separation was verified by western blot analysis of individual fractions using antibodies against ribosomal protein L22 and TUBULIN.

### RNA-seq, small RNA-seq, and CLIP-seq

Strand-directional RNA-seq libraries were prepared from total RNA (depleted of ribosomal RNA) from SPC control (shVector) and *Mov10* knockdown (shMov10-832) samples using the TruSeq Stranded Total RNA Sample Preparation kit (Illumina, USA) according to the manufacturer’s instructions. Small RNA libraries were prepared using small RNAs (size range of 15–50 nt) isolated from total RNA samples using the TruSeq Small RNA library prep kit (Illumina, USA) according to the manufacturer’s protocols. MOV10 CLIP libraries were prepared as described above. Sequencing of 3 CLIP libraries, 6 RNA-seq libraries, and 2 small RNA libraries (with triplicate samples) was performed using Illumina Hiseq2500 according to the manufacturer’s instructions.

Shanghai Biotech Co. (Shanghai, China) and Vazyme Biotech Co., Ltd. (Nanjing, China) performed bioinformatics computations. The quality of sequencing data was validated using KASAVA (version 1.8). Raw reads were pre-processed using Fastx (fastx_toolkit-0.0.13.2) to achieve clean data by filtering rRNAs and trimming adaptors. For RNA-seq and CLIP-seq, clean reads were mapped to the mouse genome (mm10) using TopHat (version 2.0.9) with a GTF file download from Ensemble database with maximum 2-base mismatch. Reads from small RNA-seq were mapped to miRBase (version 21.0) by CLC genomics workbench (version 5.5) without base mismatch.

### CLIP read pre-processing and genomic mapping

Qualified reads were sorted into three separate libraries with barcodes and then processed into clean reads to perform further analysis. All clean reads no shorter than 15 nt were mapped to the mouse reference genome (UCSC mm10 assembly). Only reads mapping uniquely to the genome were used for further analysis. Alignment data generated using Top Hat were converted from BAM into bigWig format and visualized using the UCSC genome browser. Next, we quantified the distribution of reads aligning with different genomic regions (5′-UTR, CDS, 3′-UTR and intergenic). The genomic coordinates for repeat elements were downloaded from the UCSC website (“rmsk.txt.gz”, output from “repeat masker”). The aligned reads were annotated using the GTF file <GTF_FILE> (downloaded from Ensemble). RNA expression values (FPKM) were calculated by Cufflinks v2.1.1. Scatter plots for correlations in between libraries were plotted using R script.

### CLIP read coverage across genic regions

Thirteen thousand two hundred ten MOV10-bound mRNAs met the selection criterion of FPKM ≥ 0.5 in at least two libraries, and a total of 32,617 corresponding transcripts were extracted from Ensemble. Genomic coordinates of mRNA regions (5′-UTR, CDS and 3′-UTR) were acquired from Ensemble. Each region was divided into 100 bins. The normalized sequencing depths per bin were plotted by scanning read density on every transcript across three independent CLIP libraries. To analyze the correlation of 3′-UTR length with MOV10 binding intensity, we ranked transcripts according to 3′-UTR length, followed by partitioning into terciles: long (10,872 transcripts), medium (10,872 transcripts), and short (10,873 transcripts). The normalized sequencing depth on three groups was plotted separately.

### CLIP-seq analysis on deletion-based crosslinked sites

Reverse transcription of CLIP-captured target RNA produces a deletion event at the crosslinked site. Therefore, deletion sites are by default considered sites of protein-RNA binding [67]. We extracted all MOV10 CLIP tags with mutations (including insertion, deletion or mismatch) and, using samtools and perl script, identified total 33,468 deletion sites relative to the genome from 297,083 deletion residues. Genomic information for deletion sites was acquired from mm10 GTF file (downloaded from Ensemble). To calculate nucleotide composition within 100 nt on both sides, deletion sites were set as position zero. To predict the RNA secondary structure, we assessed the possibility of base pairing and unpairing in the indicated ranges. The percentage of base pairing in each position reflects the local potential of secondary structure. This analysis was verified by an expected result of high frequency pairing of miRNA stem nucleotides compared with those on the miRNA loop.

To evaluate MOV10 binding to pre-mRNA introns, CLIP tags containing crosslinked sites falling within intronic regions were extracted [74]. Exon-intron junctions were located on a genome-wide level, and each was marked as position zero. Next, genomic windows were set around each site that consisted of the downstream 200 nt relative to the 5′ splice site and of the upstream 200 nt relative to 3′ splice site. The number of genomic crosslinked sites or crosslinked nucleotides were mapped, calculated, and plotted within these set windows. Further, ± 100 nt genomic windows flanking each exon-intron junction were scanned, and the windows with deletion sites identified on the intronic side were reserved for calculating nucleotide composition.

### CLIP-seq analysis on MOV10-bound miRNA transcripts

Genomic coordinates of miRNA hairpins were obtained from miRBase (release 21.0). Similarly to a previously reported approach [68], we examined CLIP-seq reads to identify reads located within or with an overlap of at least one nucleotide (nt) of a 100 nt window flanking either side of the miRNA hairpins. Each miRNA region was scanned to determine whether MOV10 bound to mature, pre- or pri-miRNA on the basis of read pileup patterns. We classified retained reads into five categories: in detail, category I, reads that map fully to mature miRNAs (no more than 1 nt excess sequence); category II, reads within pre-miRNA sequences that overlap with regions of the mature miRNA (at least 1 nt sequence overlapping with pre-miRNA); category III, reads mapping to pre-miRNA sequences but without overlap with mature miRNA sequence; category IV, reads that overstep the boundary of pre-miRNA sequences (an overlap of at least 1 nt sequence with pre-miRNA); and category V, reads completely outside of the pre-miRNA boundaries. A minimum of five CLIP reads was required to define each form of miRNA: mature (category I), pre-miRNA (category II plus III), and pri-miRNAs (category IV plus V). To characterize the global distribution of CLIP reads relative to the position of miRNA secondary structure, the ± 200 nt windows flanking the stem loop midpoint were extracted and divided into 30 bins for read coverage plotting.

### RNA-seq analysis on mRNA transcripts with differential 3′-UTR lengths

Six hundred eighty-nine genes with significant differences in transcript level were selected (*p* < 0.05, fold change> 1.5). The genomic coordinates of all transcripts corresponding to these genes were obtained from Ensemble. All transcripts were ranked according to the length of 3′-UTR from long to short and then divided into terciles (long, medium, and short). Transcripts levels (FPKM values) were calculated by Cufflinks v2.1.1. Transcripts without mapped reads were discarded. Expression changes were measured using log2 ratios (*Mov10* knockdown vs control cells).

### RNA-seq analysis on miRNA transcript levels

Referring to Fig. 3d, the colored parts of miRNA transcripts are matched between the left and right panels. Left: black solid lines represent pri-miRNA regions extended from stem-loop; black dashed lines represent that the two sides of a pri-miRNA region may either join or separate after initial processing; green lines represent the mature miRNA and/or its stem side; red lines represent the other stem side; and gray circles represent loop region. Right: reads mapping to ± 200 nt window flanking the miRNA hairpin, shown as staggered black lines on top of the two initial miRNA transcripts (before and after processing, respectively), were extracted from RNA-seq library. Note that these two initial transcripts cannot be separately evaluated as they would likely be overrepresented by their cognate RNA-seq reads; that both pre-miRNA (green+gray+red) and mature miRNA (green) are excluded from RNA-seq library; and that the small RNA library contains relatively short reads that are mostly mature miRNAs. The RNA-seq reads (staggered black lines) were collectively evaluated as a normalized density that roughly reflects the whole transcript level of each miRNA, equivalent to the seed levels of mature miRNA, i.e., the maximum potential of miRNA generation.

### RNA-seq analysis on alternative splicing events

To detect differentially regulated exons or isoforms at a genome-wide level, we applied a statistical model of mixture-of-isoforms (MISO) to RNA-seq data [99]. Sequencing reads were aligned to known and predicted regions for alternative splicing including exon-intron junction boundaries annotated from genome mm10. We discarded any events with less than 5 supporting reads. Specific events were identified from either the Mov10 knockdown or control libraries and then classified into five categories.

### Statistical analysis

Data are reported as mean ± SE unless otherwise noted in the figure legends. Significance between groups was determined using the two-tailed unpaired Student *T* test (*p < 0.05; **p < 0.01; ***p < 0.001).

## Additional files

Additional file 1: **Figure S1.** MOV10 antibody validation. **Figure S2.** The purity of isolated cell populations. **Figure S3.** Comparison of *Mov10* and *Mov10l1*. **Figure S4.** Assessment of the off-target effect of shRNA interference. **Figure S5.** Knockdown of endogenous *Mov10* leads to increased apoptosis in testicular tubules. **Figure S6.** MOV10 regulates gene expression. **Figure S7.** GO analysis of upregulation genes in shMov10 SPCs. **Figure S8.** Effect of *Mov10* overexpression on SPCs. **Figure S9.** MOV10 regulates miRNA precursors via 3′-UTR processing. **Figure S10.** MOV10 regulates mirtron splicing in SPCs. **Figure S11.** MOV10 regulates splicing of non-mirtron intronic miRNA in SPCs. **Figure S12.** MOV10 regulates alternative splicing in SPCs. **Figure S13.** Reproducibility and genomic mapping of MOV10 CLIP libraries. **Figure S14.** Characterization of MOV10-bound 3′-UTRs. **Figure S15.** Characterization of MOV10 binding to intronic regions. **Figure S16.** Comparison of cytoplasmic MOV10-associated proteins with those in the nucleus. **Table S1.** Mapping of MOV10 CLIP tags to the top 19 pre-pachytene piRNA clusters. **Table S3.** Genome-wide annotations of MOV10 CLIP targets. (DOCX 20239 kb)

Additional file 2: **Table S2.** Transcript IDs with different 3′-UTR length corresponding to 13,210 mRNA targets. (XLS 1387 kb)

Additional file 3: **Table S4.** CLIP reads predominantly mapping to miRNA precursors. (XLSX 157 kb)

Additional file 4: **Table S5.** MOV10-bound miRNAs and their category of maturity. (XLSX 18 kb)

Additional file 5: **Table S6.** Mass spectrometry analysis of subcellular MOV10 IP in mice testes. (XLSX 41 kb)

Additional file 6: **Table S7.** Sequences of oligonucleotides used in this study. (XLSX 18 kb)
