# Supplementary material for: Biological and RNA regulatory function of MOV10 in mammalian germ cells
Source: BMC Biol. 2019 May 14;17:39. doi: 10.1186/s12915-019-0659-z (PMC6515687; doi:10.1186/s12915-019-0659-z)
Supplement: Supplementary file 1 — Figure S1. MOV10 antibody validation. Figure S2. The purity of isolated cell populations. Figure S3. Comparison of Mov10 and Mov10l1. Figure S4. Assessment of the off-target effect of shRNA interference. Figure S5. Knockdown of endogenous Mov10 leads to increased apoptosis in testicular tubules. Figure S6. MOV10 regulates gene expression. Figure S7. GO analysis of upregulation genes in shMov10 SPCs. Figure S8. Effect of Mov10 overexpression on SPCs. Figure S9. MOV10 regulates miRNA precursors via 3′-UTR processing. Figure S10. MOV10 regulates mirtron splicing in SPCs. Figure S11. MOV10 regulates splicing of non-mirtron intronic miRNA in SPCs. Figure S12. MOV10 regulates alternative splicing in SPCs. Figure S13. Reproducibility and genomic mapping of MOV10 CLIP libraries. Figure S14. Characterization of MOV10-bound 3′-UTRs. Figure S15. Characterization of MOV10 binding to intronic regions. Figure S16. Comparison of cytoplasmic MOV10-associated proteins with those in the nucleus. Table S1. Mapping of MOV10 CLIP tags to the top 19 pre-pachytene piRNA clusters. Table S3. Genome-wide annotations of MOV10 CLIP targets. (DOCX 20239 kb) [file 12915_2019_659_MOESM1_ESM.docx]

**Additional File 1:**

Supplementary Figures and Legends S1-S16

Supplementary Tables S1 and S3

**Biological and RNA Regulatory Function of MOV10 in Mammalian Germ Cells**

Kaiqiang Fu^1,6^, Suwen Tian^2,1,6^, Huanhuan Tan^1,6^, Caifeng Wang^1,6^, Hanben Wang^1,6^, Min Wang^1^, Yuanyuan Wang^3^, Zhen Chen^4^, Yanfeng Wang^1^, Qiuling Yue^1^, Qiushi Xu^1^, Shuya Zhang^1^, Haixin Li^1^, Jie Xie^1^, Mingyan Lin^3^, Mengcheng Luo^4^, Feng Chen^5^, Lan Ye^1^, Ke Zheng^1^*

^1^State Key Laboratory of Reproductive Medicine, Nanjing Medical University, Nanjing 211166, China;

^2^Department of Preventive Medicine, Heze Medical College, Heze 274000, China;

^3^School of Basic Medical Sciences, Nanjing Medical University, Nanjing 211166, China;

^4^School of Basic Medical Sciences, Wuhan University, Wuhan 430072, China;

^5^Department of Forensic Medicine, Nanjing Medical University, Nanjing 211166, China;

^6^These authors contributed equally to this work;

*Correspondence: [kezheng@njmu.edu.cn](mailto:kezheng@njmu.edu.cn) (KZ)

Running title: MOV10 plays a critical role in male germline


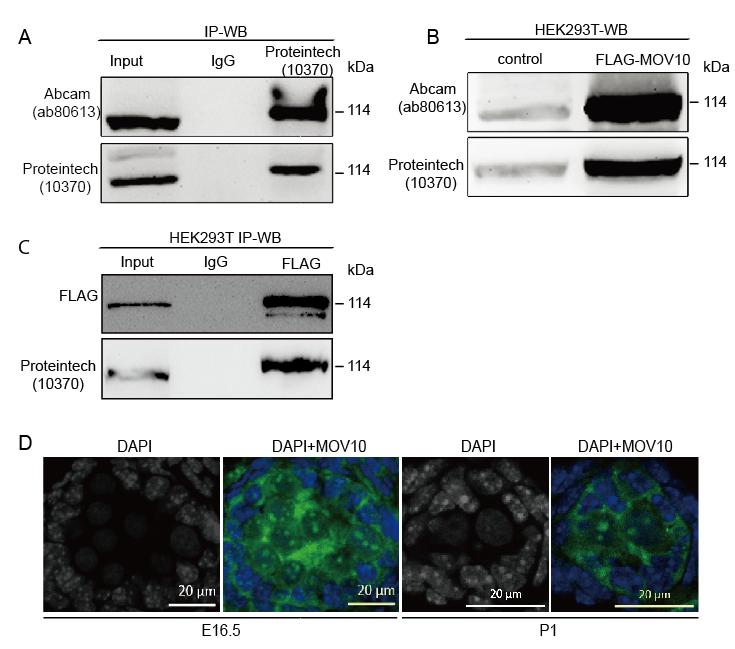


**Figure S1.** MOV10 antibody validation. (A and B) Western blot analysis of MOV10-immunoprecipitated complex from P10 mouse testis (A) and FLAG-MOV10-overexpressing HEK293T cells (B) using two commercial anti-MOV10 antibodies (from Abcam and Proteintech, respectively). The weak bands in control represent endogenous MOV10 in HEK293T cells. (C) Western blot detection of the FLAG-MOV10 fusion protein in FLAG-immunoprecipitated complex from FLAG-MOV10-overexpressing HEK293T cells using anti-FLAG and anti-MOV10. (D) Immunofluorescence of MOV10 in frozen section of testes from E16.5 (left) and P1 mice (right). DNA was counterstained with DAPI. Scale bar, 20 μm.


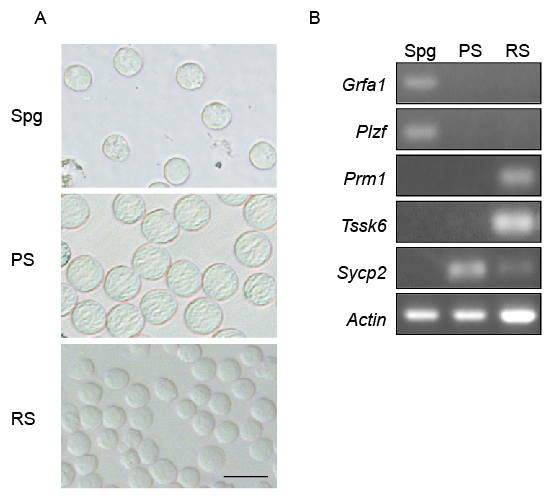


**Figure S2.** The purity of isolated cell populations. (A) The purity of germ cells isolated from mouse testis was confirmed by observation of their morphology of spermatogonia (Spg); pachytene spermatocytes (PS); round spermatids (RS). Scale bars, 20 μm. (B) The expression of key marker gene was tested by RT-PCR to further validate their purity.


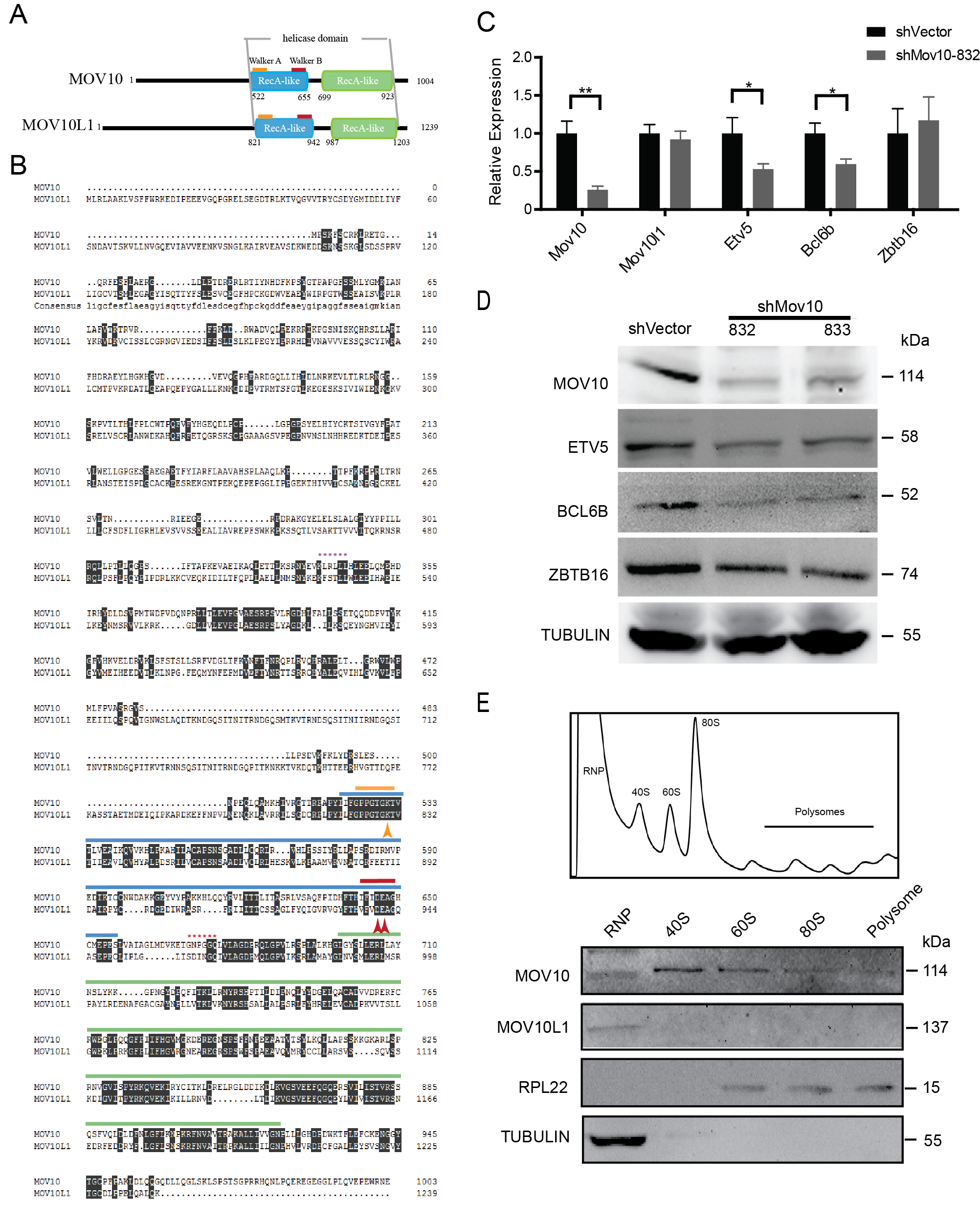
­­­

**Figure S3.** Comparison of MOV10 and MOV10L1. (A) Schematic domain structure of MOV10 and MOV10L1. The blue and green boxes represent two RecA-like domains that form a helicase core that includes the conserved N-terminal Walker A and B sequence motifs for ATP binding and ATP hydrolysis. (B) Sequence alignment of mouse MOV10 and MOV10L1. We cloned the full length CDS of *Mov10l1* (accession number: KY774831). The full length *Mov10l1* gene spans 27 exons, and encodes 1239 aa with an extended N-terminus obtained from an EST sequence (BY706660). A short form has additionally been reported that consists of only 26 exons (1187 aa). We also cloned the *Mov10* gene (accession number: NM_008619), which is shorter in length than *Mov10l1*. The consensus amino acids are shown in black. Blue and green lines identify the amino acids of Walker A and Walker B motifs. Orange and red arrowheads point to functionally critical residues of the ATP binding and ATP hydrolysis motifs. The mutations K830A and DE940AA in the long form, corresponding to K778A and DE888AA in the short form, cause loss of function of MOV10L1. Asterisks mark sequence targets of probes shMov10-832 (violet) and shMov10-833 (pink) used for knockdown experiments. Amino acids sequences were retrieved from Uniprot and were aligned using DNAMAN. (C) Quantitative PCR analysis of mRNA levels of *Mov10*, *Mov10l1* and genes important for determination of SPC fate. Data represent results from biological triplicates. (D) Western blot analysis of the protein levels of genes tested in Panel C. (E) Polysome analysis of MOV10 and MOV10L1. Following fractionation using 20-50% sucrose gradients, ribonucleoprotein particles (RNP), free ribosomal subunits (40S and 60S), 80S monosomes, and polysomes were collected for western blot. Ribosomal protein L22 (RPL22): positive control for polysome protein; TUBULIN was used as positive control for RNP.


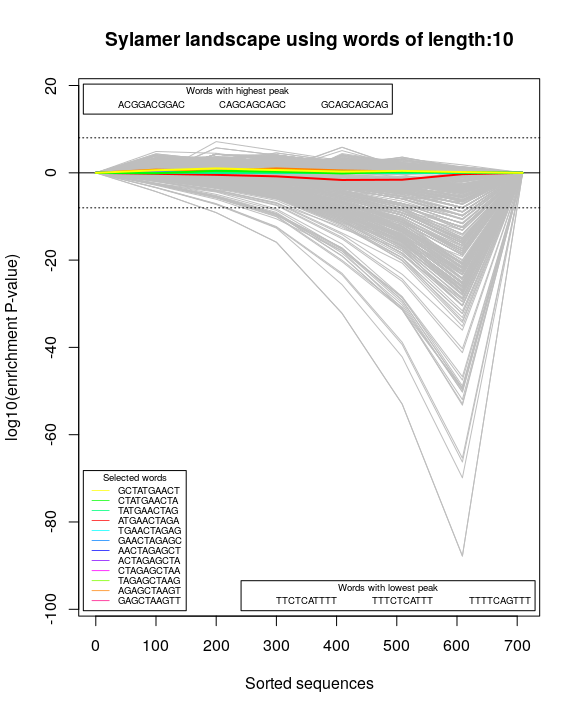


**Figure S4.** Assessment of the off-target effect of shRNA interference. Enrichment plots for a successful shRNA interference experiment in which there were no apparent off-target effects. Plot shown for 10 nt word, the x-axis represented the sorted gene-list from most downregulated (left) to most upregulated (right). The y-axis shows the hypergeometric significance for each word at each ranked gene-list set. Positive values indicate enrichment (−log10 (P-value)) and negative values, depletion (log10 (P-value)). The horizontal line represents a threshold (Bonferroni corrected p value) of 0.01.Grey lines show the profiles of all possible 10-bp words of coding sequences of differentially expressed genes, while colored lines represent all possible 10-bp words out of shRNA sequences. No words out of shRNA sequences were significantly enriched.


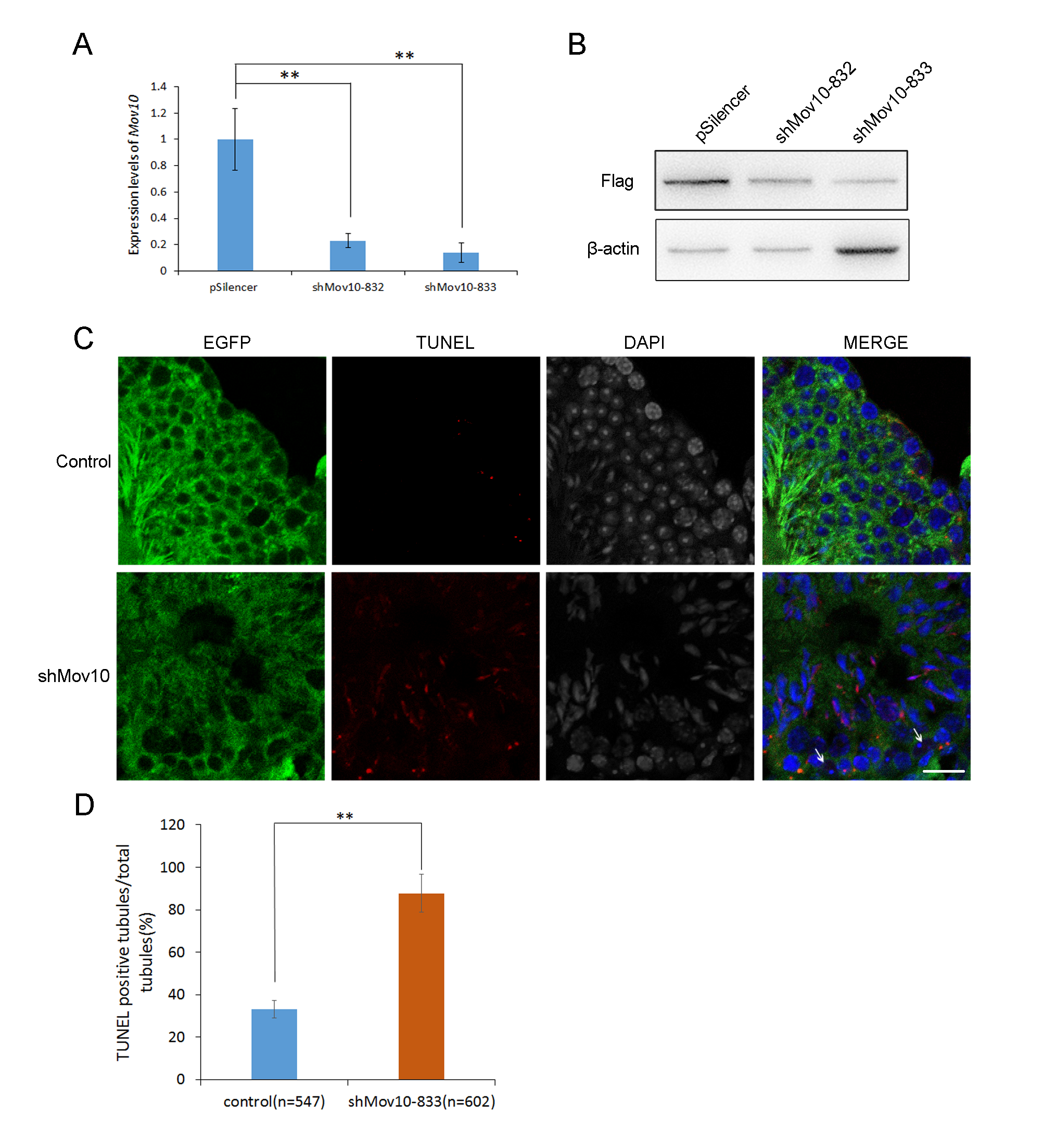


**Figure S5.** Knockdown of endogenous MOV10 leads to increased apoptosis in testicular tubules. (A, B) The knockdown efficiency of shMov10 was tested by RT-qPCR and WB in 293T cells. (C) TUNEL assay in testes sections from control and shMov10 groups. Scale bar: 20 μm. (D) Percentage of TUNEL-positive tubules in control and shMov10 groups.


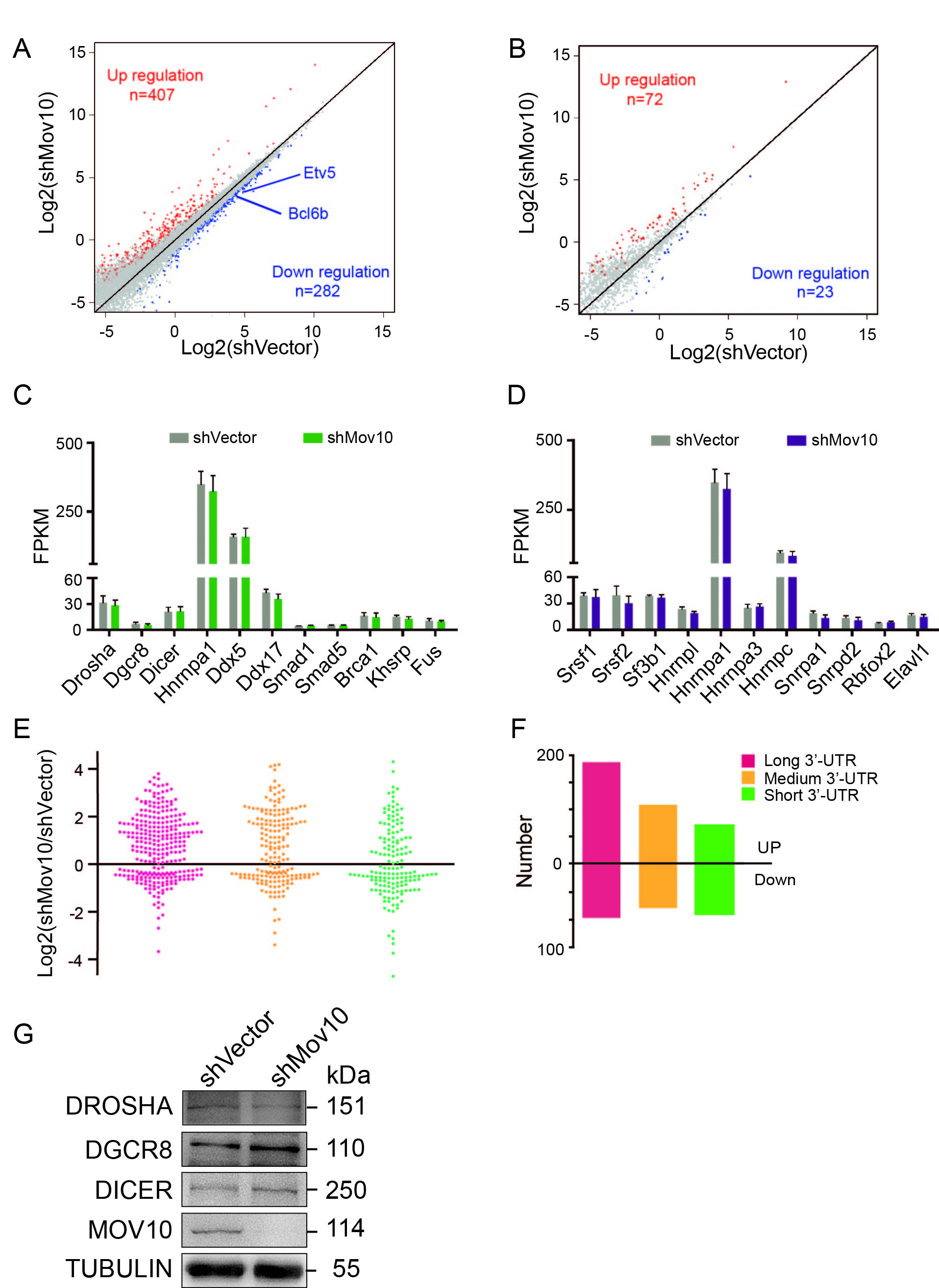


**Figure S6.** MOV10 regulates gene expression. (A) Scatter plot of mRNA expression levels from RNA-seq data. Red and blue dots represent genes with significant upregulation and downregulation, respectively (fold change>1.5, p<0.05). (B) Scatter plot of lncRNA expression levels determined by RNA-seq. Red and blue dots represent lncRNAs with significant upregulation and downregulation, respectively (fold change >1.5, p<0.05). (C and D) Analysis of RNA-seq data of genes involved in miRNA biogenesis (C) and the splicing machinery (D) after *Mov10* knockdown. The error bars represent variation among three independent CLIP libraries. (E) Differential regulation of mRNAs in a 3'-UTR length-dependent manner. Transcripts were ranked according to the length of 3’-UTR from long to short, and then divided into terciles (long, medium and short). The plot depicts the log2 fold change in transcript levels in *Mov10* knockdown vs control SPCs. (F) Quantification of upregulated and downregulated transcripts according to UTR length terciles. (G) Western blot analysis of three classical factors involved in miRNA biogenesis


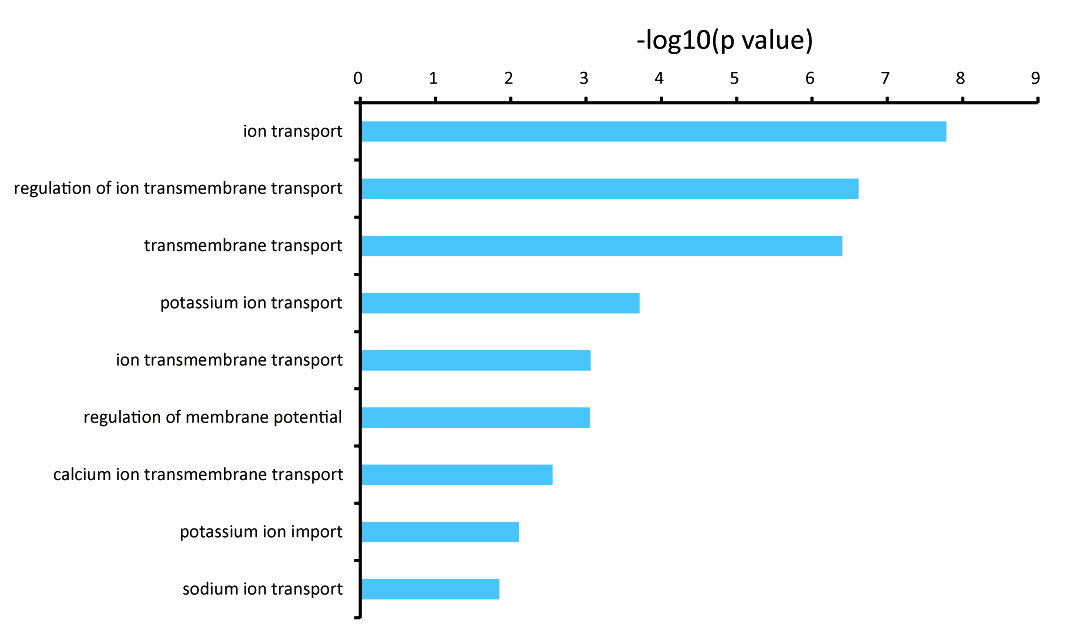


**Figure S7.** GO analysis of upregulation genes in shMov10 SPCs. The most enriched gene ontology (GO) terms among upregulated mRNAs in Mov10 knockdown SPCs.


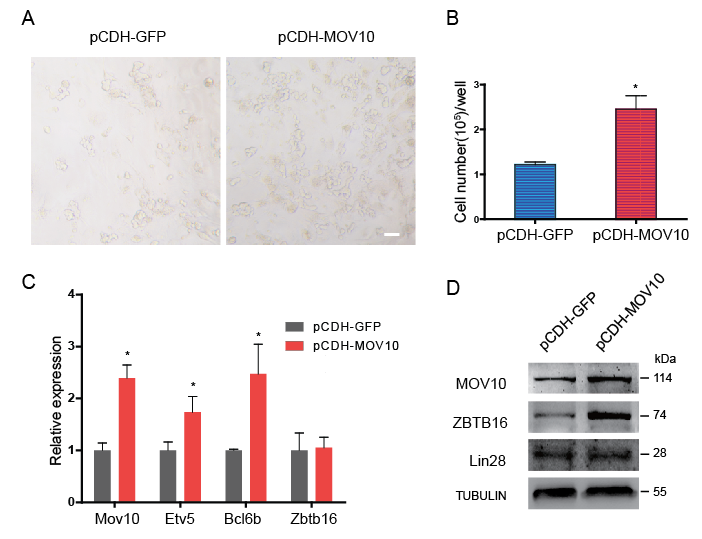


**Figure S8.** Effect of *Mov10* overexpression on SPCs. (A) *In vitro* cultured germ cells overexpressing MOV10 form large colonies. Colony size was assessed 5 days after transfection with control plasmid (GFP expression plasmid pCDH-GFP) or MOV10 expression construct (pCDH-MOV10). Scale bare, 20 µm. (B) Total cell number in SPC cultures after MOV10 overexpression. The error bars represent variation among biological triplicates. (C) Q-PCR analysis of mRNA levels of *Mov10* and genes with known role in regulation of SPC fate. Results represent data from biological triplicates. (D) Western blot analysis of the protein levels of MOV10 and ZBTB16, with LIN28 and ACTIN as controls.


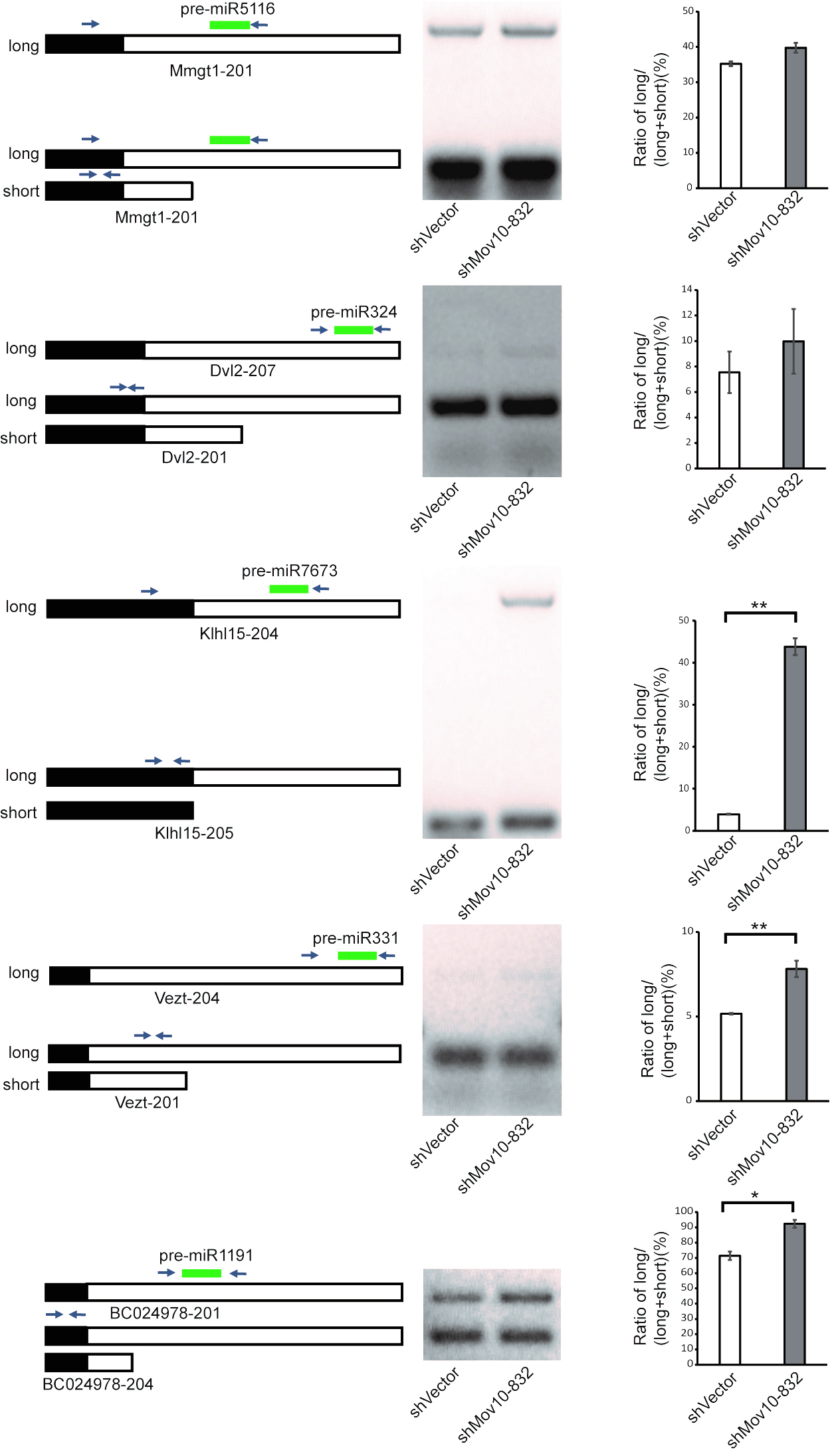


**Figure S9.** MOV10 regulates miRNA precursors via 3’-UTR processing. MiRNA processing events on 3’-UTR were assessed as described in Figure 4.

**
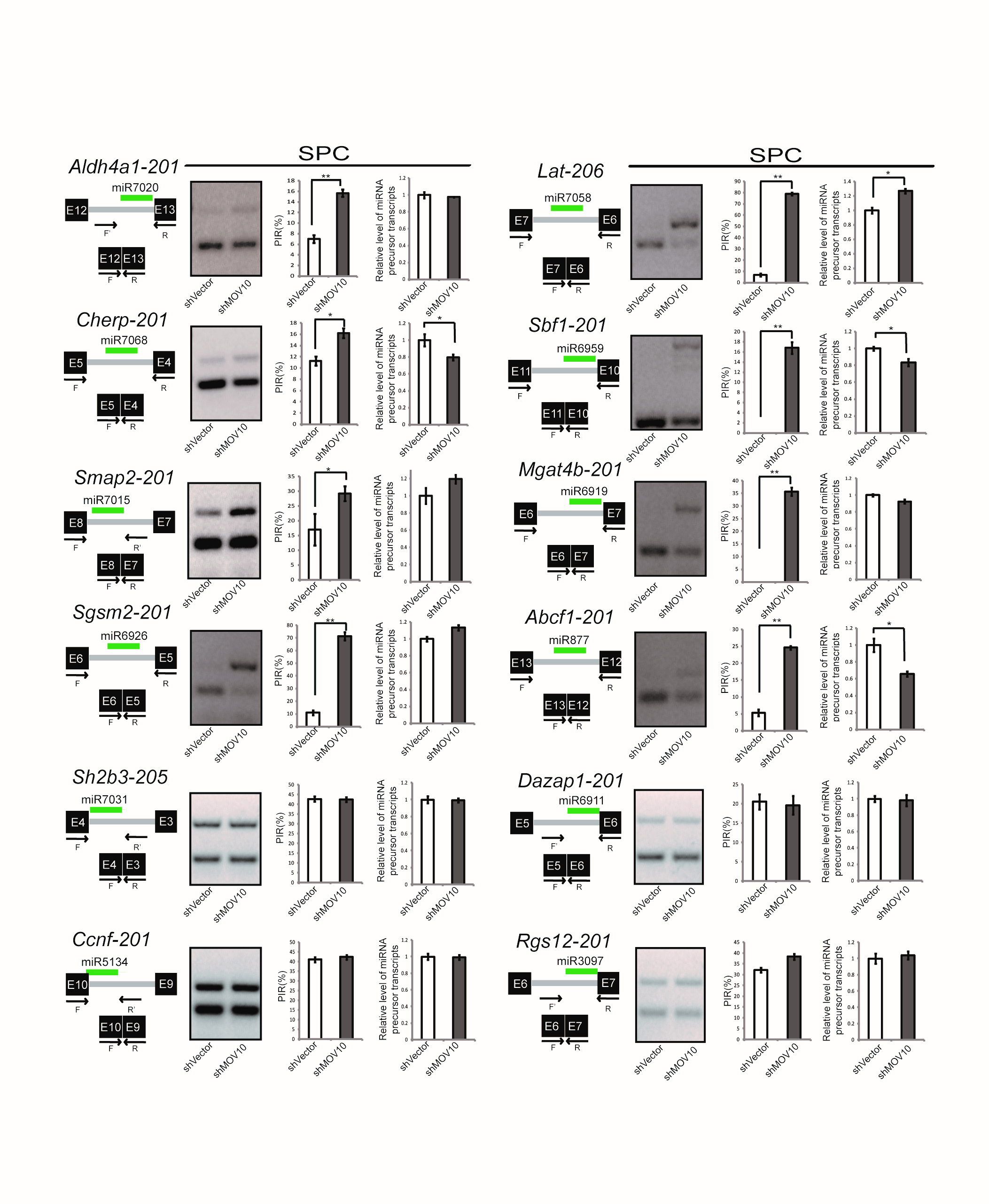
**

**Figure S10.** MOV10 regulates mirtron splicing in SPCs. Mirtron splicing events were assessed as described in Figure 4.


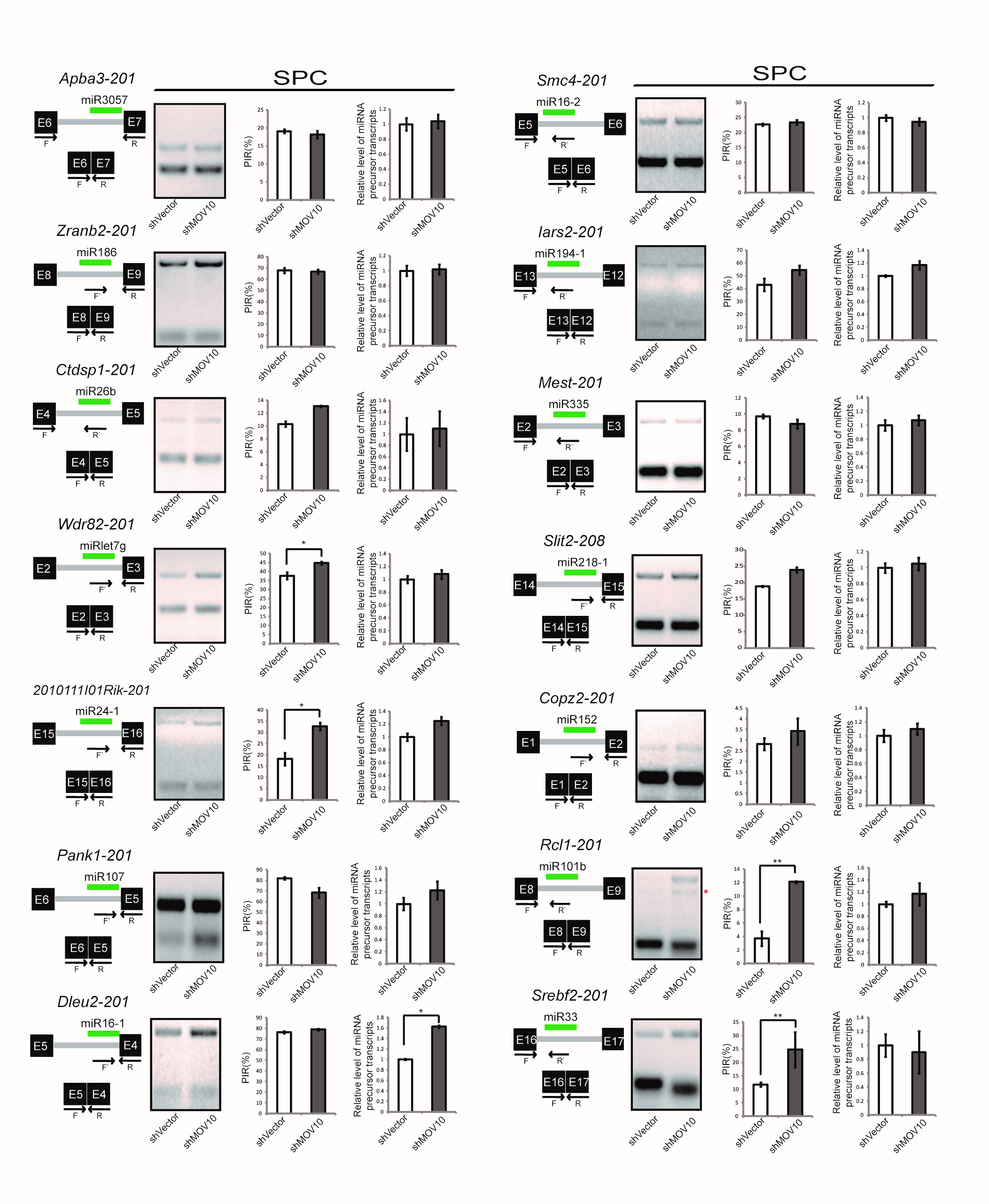


**Figure S11.** MOV10 regulates splicing of non-mirtron intronic miRNA in SPCs. Splicing events for intronic miRNAs other than mirtron were assessed as described in Figure 4. The red asterisk marks a nonspecific band.


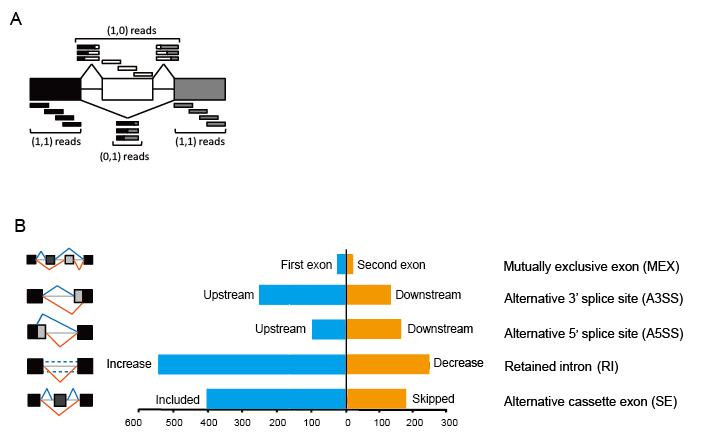


**Figure S12.** MOV10 regulates alternative splicing in SPCs. (A) Schematic of the alternative splicing analysis for MISO model. White, alternatively spliced exon; gray and black, flanking constitutive exons. RNA-seq reads aligning to the alternative exon body (white) or to splice junctions involving this exon support the inclusive isoform (1,0), whereas reads joining the two constitutive exons (black gray exon junction) support the exclusive isoform (0,1). Reads aligning to the constitutive exons are common to both isoforms (1,1). (B) Alternative splicing events were classified into 5 different categories depicted schematically to the left, with blue and orange colors representing alternate events. The bar diagram depicts the number of corresponding events identified in RNA-seq data from *Mov10* knockdown SPC.


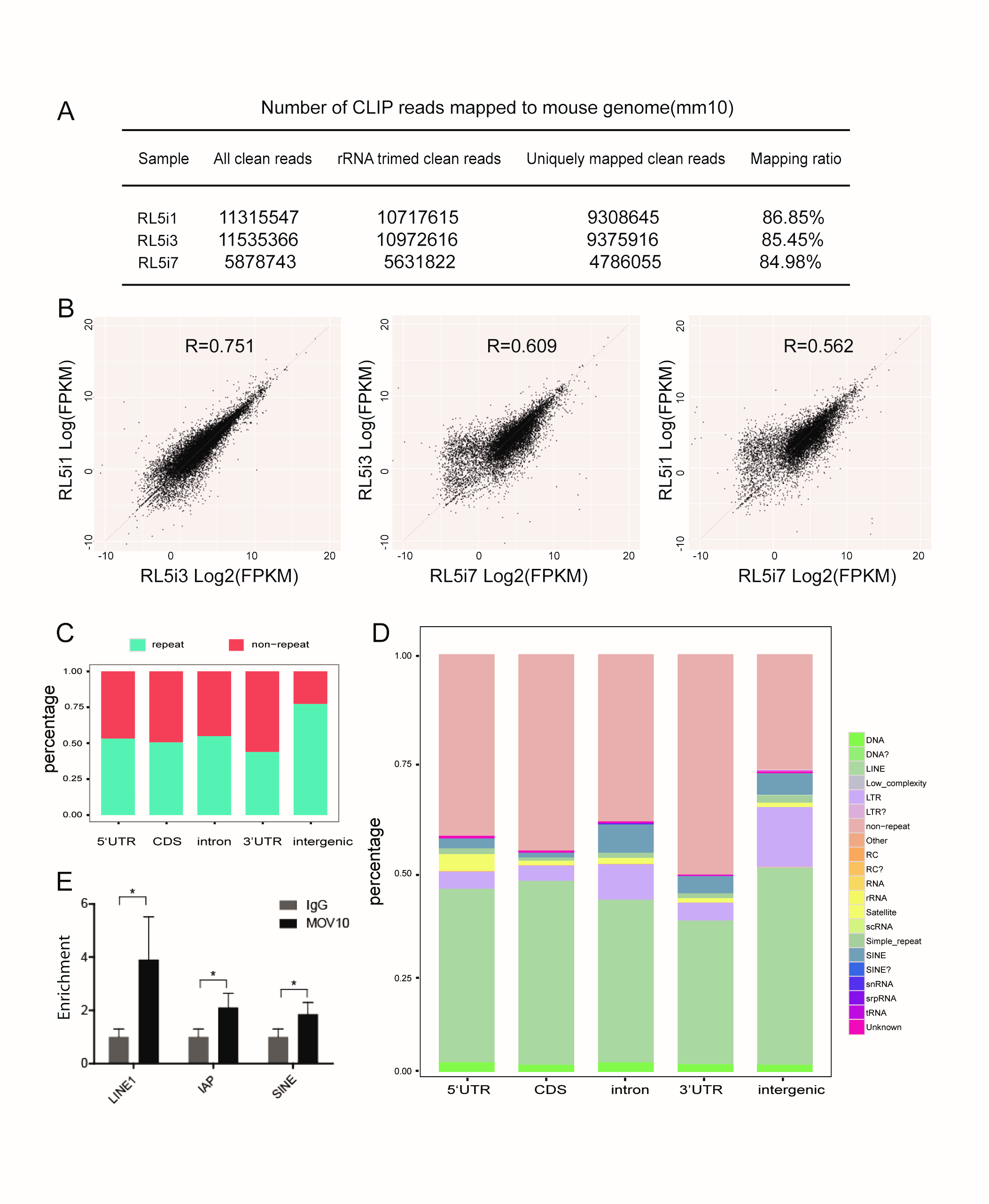


**Figure S13.** Reproducibility and genomic mapping of MOV10 CLIP libraries. (A) Mapping information of CLIP-seq data from three individual CLIP libraries. (B) Reproducibility of gene targets of MOV10 from three CLIP experiments. Each dot in the scatter plot represents log2 values of FPKM comparing the two replicates indicated on the axes. (C) Percentage of CLIP reads overlapping with repeat and non-repeat sequences within genomic regions annotated as listed below the bars. (D) Percentage of CLIP tags mapping to various types of repeat and non-repeat sequence annotations. Genic repeats may reflect embedded repeats in genes because the origin of a repeat-derived tag cannot be traced back to mRNAs. (E) Validation of MOV10-bound LINE1, IAP (LTR) and SINE by RIP-qPCR. Results from biological triplicates were analyzed.


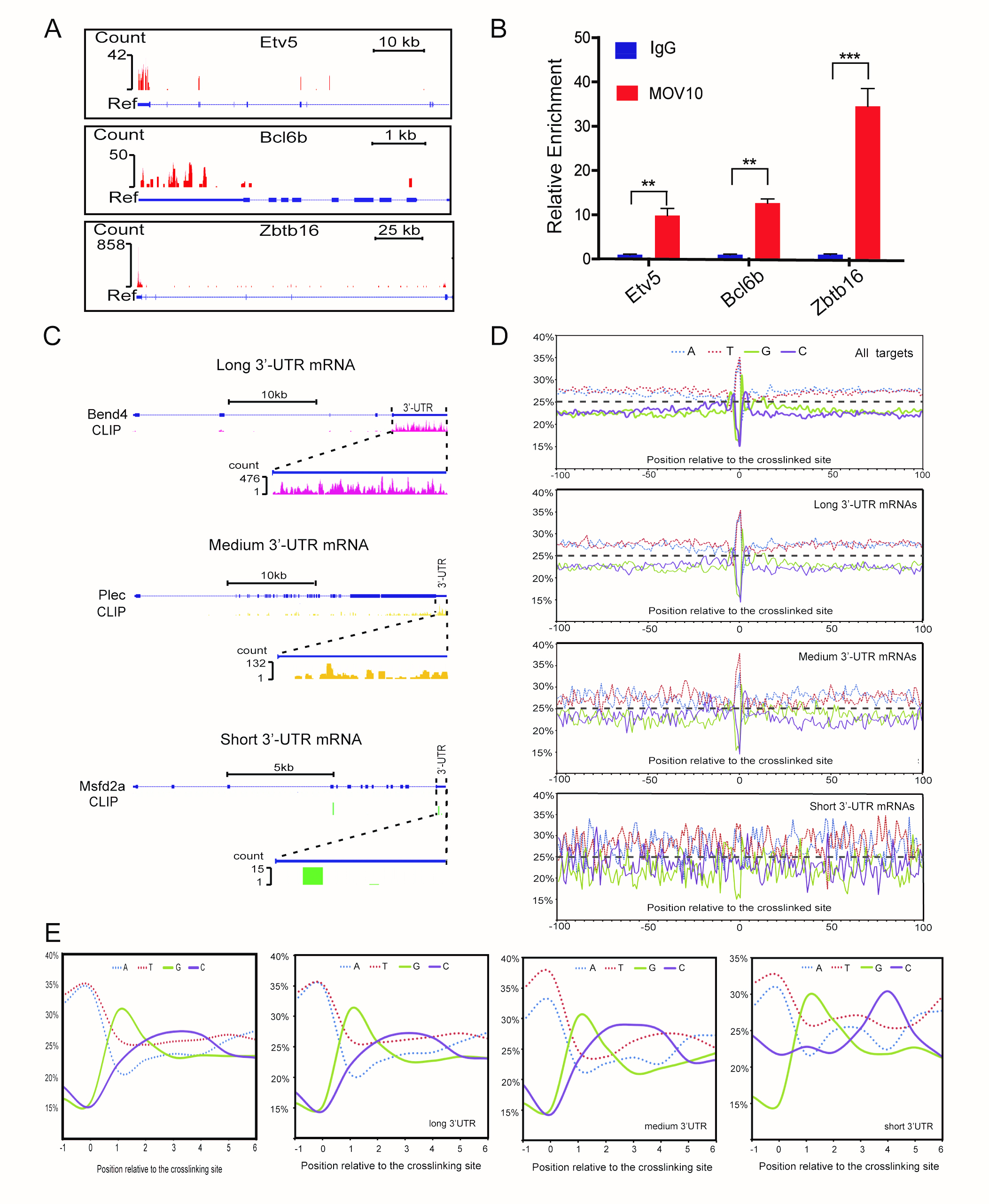


**Figure S14.** Characterization of MOV10-bound 3’-UTRs. (A) Visualization of MOV10 CLIP reads on *Etv5*, *Bcl6b* and *Zbtb16* transcripts using the UCSC genome browser. All 3 genes are transcribed from the (-) strand. MOV10 CLIP reads predominantly align with the 3’-UTRs. (B) Validation of MOV10 binding to *Etv5*, *Bcl6b* and *Zbtb16* mRNAs by RIP-qPCR. Results from biological triplicates were analyzed. (C) UCSC visualization of three representative genes containing long, medium and short 3’-UTRs. (D) Nucleotide composition per position within ±100 nt from crosslinked sites. Nucleotide percentage at each position was calculated from all unique mapped reads. Separate graphs are shown for all CLIP targets and for mRNA targets with long, medium, and short 3’-UTR. (E) Line graphs are separately shown of nucleotide content per position from upstream 1 nt to downstream 6 nt relative to crosslinked sites.


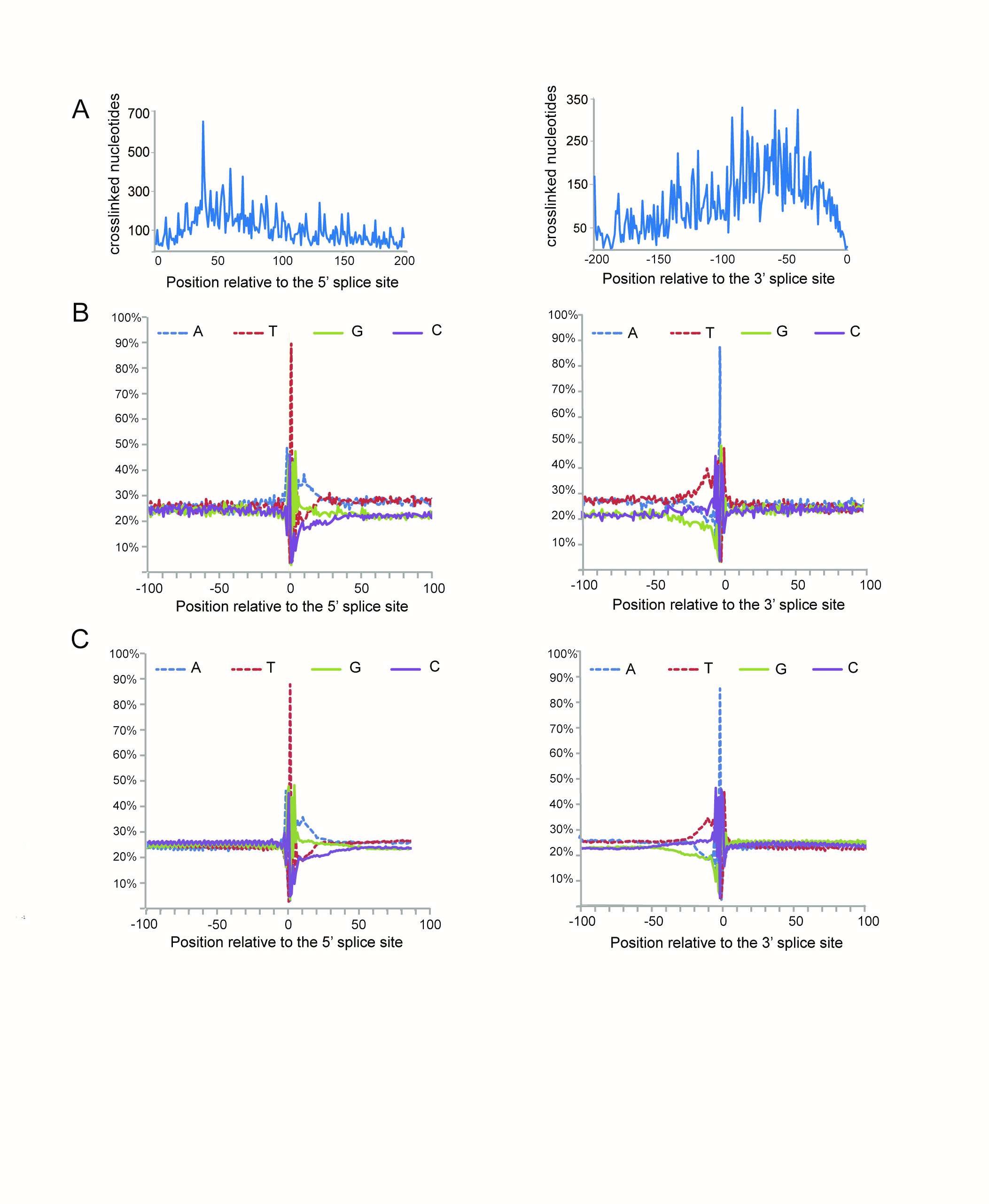


**Figure S15.** Characterization of MOV10 binding to intronic regions. (A) MOV10-crosslinked positions within introns. The plot shows the distribution of genomic crosslinked nucleotides within intronic regions upstream and downstream of the splice site. A genomic crosslinked nucleotide was defined as a nucleotide absent in a CLIP tag versus the corresponding genome sequence. Crosslinked nucleotides corresponding to the same deletion are presented cumulatively. (B and C) Genomic nucleotide composition per site within ±100 nt from the 5’ and 3’ splice sites with intronic regions bound by MOV10 (B), compared with that from the splice sites across the whole genome (C).


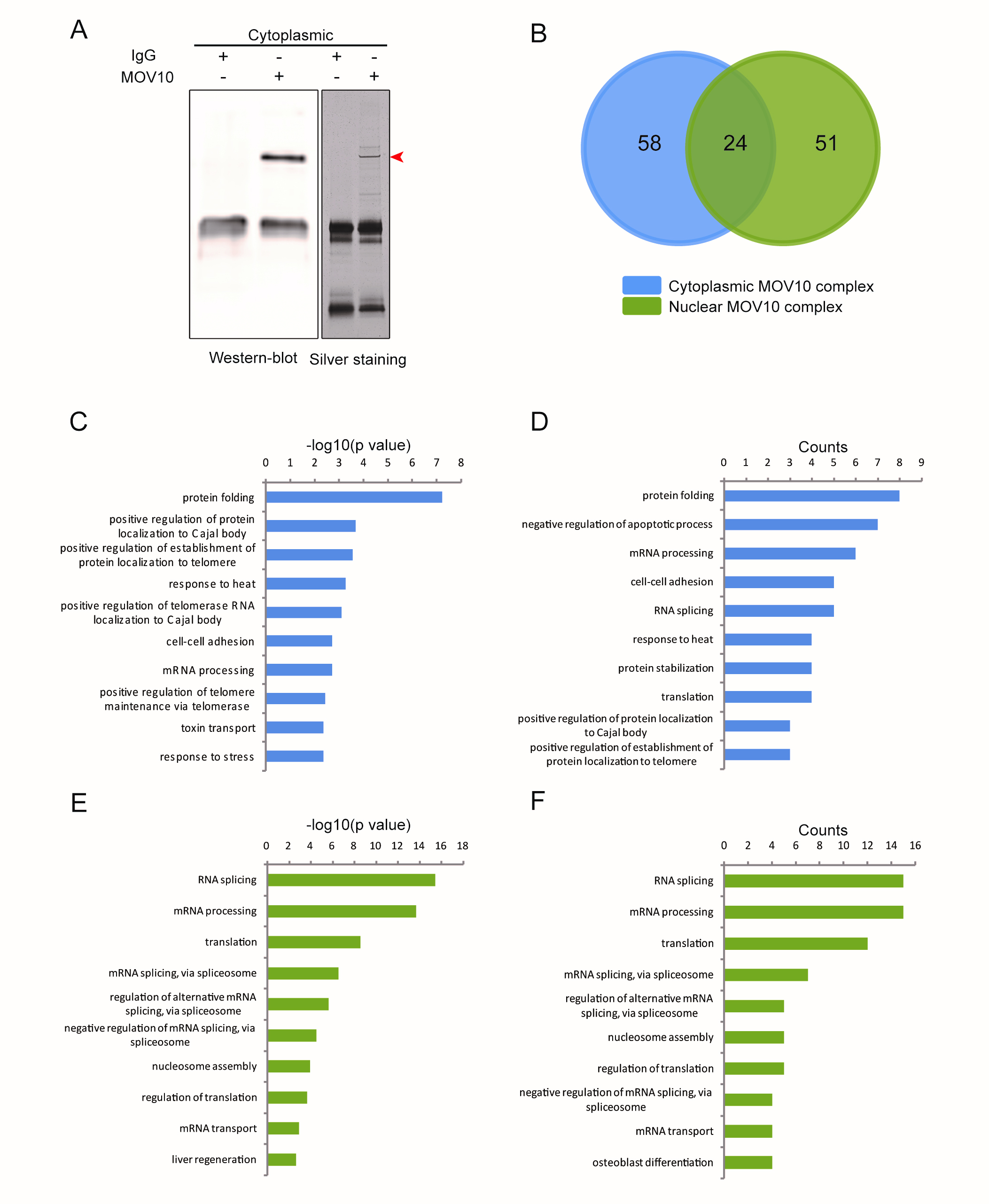


**Figure S16.** Comparison of cytoplasmic MOV10-associated proteins with those in the nucleus. (A) Western blot and silver staining of the MOV10 IP complex from cytoplasmic lysate. (B) Venn Diagram showing the numbers of cytoplasmic MOV10 and nuclear MOV10-associated proteins. The overlap (24 proteins) represent MOV10-associated proteins that could be nucleocytoplasmic. (C and D) Gene ontology analysis of the 58 unique components from the cytoplasmic MOV10-IP complex, with top 10 associations shown as p value (C) or counts (D). (E and F) Gene ontology analysis of the 51 unique components from the nuclear MOV10-IP complex, with top 10 associations shown as p value (E) or counts (F).

**Table S1.** Mapping of MOV10 CLIP tags to the top 19 pre-pachytene piRNA clusters

|  | Position（mm10） | Strand | Cluster coverage region | CLIP reads |
| --- | --- | --- | --- | --- |
| cluster1 | chr7:6573318-  6652439 | - | intergenic | partial |
| cluster2 | chr1:132023674-  132032484 | + | 3'UTR, intron, exon | full |
| cluster3 | chr7:75768979-  75782325 | - | intron, exon, 3'UTR | full |
| cluster4 | chr1:156641076-  156653920 | + | intergenic, 3'UTR, exon, intron | partial |
| cluster5 | chr10:83360291-  83457267 | + | 3'UTR | partial |
| cluster6 | chr8:117205288-  117215758 | + | intergenic | full |
| cluster7 | chr3:95181852-  95190581 | - | 3'UTR, exon, intron | partial |
| cluster8 | chr15:103191979-  103199744 | - | 3'UTR | full |
| cluster9 | chr9:114507327-  114513740 | - | intergenic, 3'UTR, exon | full |
| cluster10 | chr4:56867119-  56880202 | - | 3'UTR | full |
| cluster11 | chr11:79684253-  79697847 | + | 3'UTR, exon, intron | partial |
| cluster12 | chr1:118677480-  118685051 | + | 3'UTR | full |
| cluster13 | chr4:108175260-  108181861 | - | intergenic | full |
| cluster14 | chr5:67392260-  67400272 | - | 3'UTR, exon, intron | partial |
| cluster15 | chr4:135412459-  135417954 | - | 3'UTR, exon, intron | partial |
| cluster16 | chr17:83574352-  83601953 | - | intergenic, 3'UTR, exon, intron | partial |
| cluster17 | chr15:102279895-  102298980 | + | intergenic, 3'UTR, exon, intron | full |
| cluster18 | chr6:83362975-  83369273 | - | 5'UTR, exon, intron, 3'UTR | full |
| cluster19 | chr16:17257386-  17263222 | + | exon, 3'UTR | partial |

**Table S3.** Genome-wide annotations of MOV10 CLIP targets

| Classification | Reproducible_0.5 | Ratio |
| --- | --- | --- |
| 3’_overlapping_ncrna | 0 | 0.00% |
| antisense | 450 | 2.49% |
| bidirectional_promoter_lncrna | 3 | 0.02% |
| IG_C_gene | 0 | 0.00% |
| IG_C_pseudogene | 0 | 0.00% |
| IG_V_gene | 1 | 0.01% |
| IG_V_pseudogene | 2 | 0.01% |
| lincRNA | 495 | 2.74% |
| macro_lncRNA | 0 | 0.00% |
| miRNA | 324 | 1.79% |
| misc_RNA | 103 | 0.57% |
| Mt_rRNA | 2 | 0.01% |
| Mt_tRNA | 18 | 0.10% |
| polymorphic_pseudogene | 7 | 0.04% |
| processed_pseudogene | 2009 | 11.12% |
| processed_transcript | 236 | 1.31% |
| protein_coding | 13210 | 73.09% |
| pseudogene | 51 | 0.28% |
| ribozyme | 6 | 0.03% |
| rRNA | 67 | 0.37% |
| scaRNA | 5 | 0.03% |
| sense_intronic | 39 | 0.22% |
| sense_overlapping | 4 | 0.02% |
| snoRNA | 102 | 0.56% |
| snRNA | 391 | 2.16% |
| TEC | 293 | 1.62% |
| TR_C_gene | 1 | 0.01% |
| TR_J_gene | 1 | 0.01% |
| TR_V_gene | 4 | 0.02% |
| TR_V_pseudogene | 1 | 0.01% |
| transcribed_processed_pseudogene | 76 | 0.42% |
| transcribed_unitary_pseudogene | 1 | 0.01% |
| transcribed_unprocessed_pseudogene | 34 | 0.19% |
| translated_unprocessed_pseudogene | 0 | 0.00% |
| unitary_pseudogene | 7 | 0.04% |
| unprocessed_pseudogene | 130 | 0.72% |

Reproducible_0.5: FPKM≥0.5 and reproducible in at least two libraries.
